# Association of Modifiable Health Conditions and Social Determinants of Health With Late Mortality in Survivors of Childhood Cancer

**DOI:** 10.1001/jamanetworkopen.2022.55395

**Published:** 2023-02-10

**Authors:** Matthew J. Ehrhardt, Qi Liu, Stephanie B. Dixon, Eric Caron, Debbie Redd, Kyla Shelton, I-Chan Huang, Nickhill Bhakta, Kirsten K. Ness, Daniel A. Mulrooney, Tara M. Brinkman, Wassim Chemaitilly, Angela Delaney, Gregory T. Armstrong, Deo Kumar Srivastava, Alia Zaidi, Leslie L. Robison, Yutaka Yasui, Melissa M. Hudson

**Affiliations:** 1 Department of Oncology, St Jude Children’s Research Hospital, Memphis Tennessee; 2 Department of Epidemiology and Cancer Control, St Jude Children’s Research Hospital, Memphis, Tennessee; 3 Department of Public Health Sciences, University of Alberta, Alberta, Canada; 4 Department of Global Pediatric Oncology, St Jude Children’s Research Hospital, Memphis Tennessee; 5 University of Pittsburgh Medical Center, Children’s Hospital of Pittsburgh, Pittsburgh, Pennsylvania; 6 Department of Pediatric Medicine, St Jude Children’s Research Hospital, Memphis, TN; 7 Department of Epidemiology and Cancer Control, St Jude Children’s Research Hospital, Memphis, TN; 8 Department of Biostatistics, St Jude Children’s Research Hospital, Memphis, Tennessee

## Abstract

**Importance:**

Associations between modifiable chronic health conditions (CHCs), social determinants of health, and late mortality (defined as death occurring ≥5 years after diagnosis) in childhood cancer survivors are unknown.

**Objective:**

To explore associations between modifiable CHCs and late mortality within the context of social determinants of health.

**Design, Setting, and Participants:**

This longitudinal cohort study used data from 9440 individuals who were eligible to participate in the St Jude Lifetime Cohort (SJLIFE), a retrospective cohort study with prospective clinical follow-up that was initiated in 2007 to characterize outcomes among childhood cancer survivors. Eligible individuals had survived 5 or more years after childhood cancer diagnosis, were diagnosed between 1962 and 2012, and received treatment at St Jude Children’s Research Hospital were included in mortality estimates. A total of 3407 adult SJLIFE participants (aged ≥18 years) who completed an on-campus assessment were included in risk factor analyses. Vital status, date of death, and cause of death were obtained by linkage with the National Death Index (coverage from inception to December 31, 2016). Deaths occurring before inception of the National Death Index were obtained from the St Jude Children’s Research Hospital Cancer Registry. Data were analyzed from June to December 2022.

**Exposures:**

Data on treatment exposures and causes of death were abstracted for individuals who were eligible to participate in the SJLIFE study. Information on modifiable CHCs (dyslipidemia, hypertension, diabetes, underweight or obesity, bone mineral deficiency, hypogonadism, hypothyroidism, and adrenal insufficiency, all graded by the modified Common Terminology Criteria for Adverse Events), healthy lifestyle index (smoking status, alcohol consumption, body mass index [calculated as weight in kilograms divided by height in meters squared], and physical activity), area deprivation index (ADI; which measures neighborhood-level socioeconomic disadvantage), and frailty (low lean muscle mass, exhaustion, low energy expenditure, slowness, and weakness) was obtained for participants.

**Main Outcomes and Measures:**

National Death Index causes of death were used to estimate late mortality using standardized mortality ratios (SMRs) and 95% CIs, which were calculated based on US mortality rates. For the risk factor analyses (among participants who completed on-campus assessment), multivariable piecewise exponential regression analysis was used to estimate rate ratios (RRs) and 95% CIs for all-cause and cause-specific late mortality.

**Results:**

Among 9440 childhood cancer survivors who were eligible to participate in the SJLIFE study, the median (range) age at assessment was 27.5 (5.3-71.9) years, and the median (range) duration of follow-up was 18.8 (5.0-58.0) years; 55.2% were male and 75.3% were non-Hispanic White. Survivors experienced increases in all-cause mortality (SMR, 7.6; 95% CI, 7.2-8.1) and health-related late mortality (SMR, 7.6; 95% CI, 7.0-8.2). Among 3407 adult SJLIFE participants who completed an on-campus assessment, the median (range) age at assessment was 35.4 (17.9-69.8) years, and the median (range) duration of follow-up was 27.3 (7.3-54.7) years; 52.5% were male and 81.7% were non-Hispanic White. Models adjusted for attained age, sex, race and ethnicity, age at diagnosis, treatment exposures, household income, employment status, and insurance status revealed that having 1 modifiable CHC of grade 2 or higher (RR, 2.2; 95% CI, 1.2-4.0; *P* = .01), 2 modifiable CHCs of grade 2 or higher (RR, 2.6; 95% CI, 1.4-4.9; *P* = .003), or 3 modifiable CHCs of grade 2 or higher (RR, 3.6; 95% CI, 1.8-7.1, *P* < .001); living in a US Census block with an ADI in the 51st to 80th percentile (RR, 5.5; 95% CI, 1.3-23.5; *P* = .02), an ADI in the 81st to 100th percentile (RR, 8.7; 95% CI, 2.0-37.6; *P* = .004), or an unassigned ADI (RR, 15.7; 95% CI, 3.5-70.3; *P* < .001); and having frailty (RR, 2.3; 95% CI, 1.3-3.9; *P* = .004) were associated with significant increases in the risk of late all-cause death. Similar associations were observed for the risk of late health-related death (1 modifiable CHC of grade ≥2: RR, 2.2 [95% CI, 1.1-4.4; *P* = .02]; 2 modifiable CHCs of grade ≥2: RR, 2.5 [95% CI, 1.2-5.2; *P* = .01]; 3 modifiable CHCs of grade ≥2: RR, 4.0 [95% CI, 1.9-8.4; *P* < .001]; ADI in 51st-80th percentile: RR, 9.2 [95% CI, 1.2-69.7; *P* = .03]; ADI in 81st-100th percentile: RR, 16.2 [95% CI, 2.1-123.7; *P* = .007], unassigned ADI: RR, 27.3 [95% CI, 3.5-213.6; *P* = .002]; and frailty: RR, 2.3 [95% CI, 1.2-4.1; *P* = .009]).

**Conclusions and Relevance:**

In this cohort study of childhood cancer survivors, living in a Census block with a high ADI and having modifiable CHCs were independently associated with an increased risk of late death among survivors of childhood cancer. Future investigations seeking to mitigate these factors will be important to improving health outcomes and developing risk-stratification strategies to optimize care delivery to childhood cancer survivors.

## Key Points

**Question:**

Are modifiable chronic health conditions (CHCs) and social determinants of health associated with late mortality (death occurring ≥5 years after diagnosis) in childhood cancer survivors?

**Findings:**

In this cohort study of 9440 eligible individuals who survived 5 or more years after diagnosis of childhood cancer, survivors were at 7-fold significantly increased risk of all-cause and health-related death, with 3407 study participants having 10 or more CHCs of grade 1 to 4 or 3 or more CHCs of grade 3 to 4 at baseline at the highest risk. Having 1 or more modifiable CHCs of grade 2 or higher, living in a US Census block with the most disadvantaged area deprivation index, and having frailty were associated with significant increases in all-cause and health-related late mortality.

**Meaning:**

These findings suggest that mitigation of socioeconomic factors and modifiable CHCs will be important to improving health outcomes and developing risk-stratification strategies to optimize care delivery to survivors of childhood cancer.

## Teaser

This cohort study uses data from the St Jude Lifetime Cohort to assess the association of modifiable chronic health conditions and socioeconomic factors with late mortality among adult survivors of childhood cancer.

## Introduction

Despite progress in treatments for childhood cancer, long-term survivors remain at increased risk of late death (defined as death occurring ≥5 years after cancer diagnosis) compared with the general population.^1,2,3,4,5^ While reductions in treatment intensity have played a role in substantial decreases in late mortality,^1^ the increasing population of long-term cancer survivors remains at high risk of premature death, largely associated with subsequent neoplasms and cardiac and pulmonary causes.^6^

Beyond treatment-associated risks, modifiable risk factors provide potential targets for interventions to reduce late mortality in survivors. Traditional cardiovascular risk factors (ie, hypertension, diabetes, dyslipidemia, and obesity) have been found to potentiate the risk of cardiac events in survivors exposed to anthracyclines and/or chest radiotherapy higher than that expected from purely additive risks.^7^ These factors, in addition to behavioral and lifestyle factors (eg, physical activity and tobacco use) and other health conditions (eg, frailty), may further impact late mortality in survivors.

Individual-level disadvantages, such as lower educational attainment,^8^ unemployment or employment in lower-skilled jobs,^9,10,11^ inadequate insurance coverage,^12^ and lower income^9,11^ are prevalent in childhood cancer survivors compared with peers. In addition, population-level measures of deprivation, such as the area deprivation index (ADI), which accounts for educational level, employment status, housing quality, and poverty measures at the Census block level (containing 600-3000 individuals per block), have been validated for a range of adverse health events in the general population^13,14,15,16,17^; however, associations between prevalent social determinants of health, ADI, and late mortality in childhood cancer survivors remain unknown. In this longitudinal cohort study, we aimed to explore the association between potentially modifiable health conditions and cancer mortality within the context of social determinants of health.

## Methods

### Study Design

We used data from the St Jude Lifetime Cohort (SJLIFE), a retrospective cohort study with prospective clinical follow-up and ongoing accrual that was initiated in 2007 to characterize outcomes of childhood cancer survivors; the design and methods used in the SJLIFE study have previously been reported.^18,19^ Eligible individuals were diagnosed and treated for childhood cancer at St Jude Children’s Research Hospital (SJCRH) between 1962 and 2012 and survived 5 or more years after diagnosis. Study participation included a comprehensive clinical, survey, and laboratory assessment at SJCRH, repeated at approximately 5-year intervals after study enrollment. The study was reviewed and approved by the SJCRH Institutional Review Board, and all participants provided written informed consent. This study followed the Strengthening the Reporting of Observational Studies in Epidemiology (STROBE) reporting guideline for cohort studies.^20^

### Study Participants

To assess late mortality without bias, we included all of the 9440 5-year survivors eligible for the SJLIFE study in the overall mortality analyses, regardless of their participation in SJLIFE or their vital status. To evaluate associations between modifiable health conditions, social determinants, and late mortality, analyses were restricted to the 3407 adult participants (aged ≥18 years) in the SJLIFE study who were evaluated on campus (hereinafter referred to as on-campus participants) and for whom data on modifiable health conditions and social determinants were available.

### Study Procedures

Trained research staff abstracted data on treatment exposures (information on cumulative chemotherapy and region-specific radiotherapy doses obtained by A.Z.) and vital status (information from the National Death Index [NDI] obtained by E.C., D.R., and K.S.; medical reports obtained by A.Z; cancer registry follow-up; and next-of-kin contact) from the medical records of individuals eligible for participation in the SJLIFE study.

Additional medical record validation of health events occurring between their last SJCRH follow-up and SJLIFE on-campus clinical or laboratory assessment was conducted for on-campus participants.^18^ Chronic health conditions (CHCs) were graded using a modified version of the National Cancer Institute Common Terminology Criteria for Adverse Events, version 4.03, which is routinely used across survivor cohorts.^19^ From 168 routinely graded CHCs, we selected potentially modifiable conditions (ie, dyslipidemia,^21^ hypertension,^22^ diabetes,^23^ underweight or obesity,^24^ bone mineral deficiency,^25^ hypogonadism,^26,27^ hypothyroidism,^28^ and adrenal insufficiency^29^) that are associated with increased mortality in the general population and for which treatment and interventions were readily available and could conceivably alter the risk of late death (ie, suggesting modifiable conditions).

The last-reported residential addresses of on-campus participants were geocoded to determine neighborhood-level socioeconomic status (SES) by US Census blocks using the ADI, a composite measure derived from components of the American Community Survey reflective of 17 neighborhood-level SES measures (eg, household income, employment status, and educational level).^13,14,15,16,17^ Each block was previously assigned a national percentile, ranking minimum SES disadvantage in the 1st percentile and maximum disadvantage in the 100th percentile. For 4.9% of on-campus participants, the ADI was designated as unassigned due to the unavailability of geocoding information (eg, post office boxes or international addresses).

On-campus participants were assigned a healthy lifestyle index score using a composite of smoking status (ever or current vs never), alcohol consumption (heavy or risky drinking [>4 drinks/d or >14 drinks/wk for men and >3 drinks/d or >7 drinks/wk for women] vs no heavy or risky drinking), physical activity (meeting Centers for Disease Control and Prevention [CDC] guidelines [ie, ≥75 minutes of vigorous activity or ≥150 minutes of moderate and/or vigorous combined activity] vs not meeting CDC guidelines), and body mass index (calculated as weight in kilograms divided by height in meters squared; 18.5 to <25.0 vs other body mass index categories). Each domain of the healthy lifestyle index was scored 1 if healthy and 0 if otherwise, with scores of 4 assigned to those considered healthy in all 4 domains and 0 assigned to those considered unhealthy in all 4 domains. Frailty was defined using the Fried criteria^30^ (eTable 1 in Supplement 1). Participants with 2 of the following conditions were defined as having prefrailty: (1) low muscle mass, (2) weakness, (3) slow walking speed, (4) self-reported exhaustion, or (5) low energy expenditure. Participants with 3 or more of these conditions were defined as having frailty.

### Outcomes

The primary outcome was all-cause and cause-specific late mortality. Vital status, date of death, and cause of death were obtained by linkage with the National Death Index (coverage from inception to December 31, 2016).^18^ Deaths occurring before inception of the National Death Index were obtained from the SJCRH Cancer Registry. Data were analyzed from June to December 2022. Probabilistic matching^19^ was performed followed by application of the CDC National Program for Cancer Registries data linkage process.^20^ Manual review of death records from the NDI, supplemented by available medical records, was conducted to establish a centrally determined cause of death for comparison.^1^ Causes of death were categorized as (1) recurrence or progression of primary cancer, (2) health-related (including nonrecurrence and nonexternal causes of death [eg, subsequent neoplasms and cardiac causes]), or (3) external (eg, accidental causes).

### Statistical Analysis

Late mortality was evaluated using time-to-event methods, with at-risk status starting 5 years after cancer diagnosis and ending at the earlier of death (event of interest) or the date of NDI coverage (censoring). We estimated all-cause cumulative mortality by years since diagnosis for each group of causes of death, treating the other causes as competing risks. Cumulative mortality curves comparing NDI-assigned and SJLIFE-assigned causes of death began after the first SJLIFE on-campus assessment (hereinafter referred to as baseline) for all adult on-campus participants, stratified by the number of CHCs at baseline.

For SJLIFE-eligible individuals, we used NDI causes of death to calculate (1) mortality rates per 1000 person-years by 5-year follow-up intervals; (2) standardized mortality ratios (SMRs) and 95% CIs using age-specific, sex-specific, and calendar year–specific US mortality rates from the National Center for Health Statistics; and (3) cause-specific SMRs for deaths due to subsequent neoplasms, cardiac causes, pulmonary causes, external causes, and other causes (excluding recurrence and progression) according to the *International Statistical Classification of Diseases and Related Health Problems, Tenth Revision *(*ICD-10*). For deaths occurring before 1999, causes of death from the *International Classification of Diseases, Ninth Revision* were converted to the corresponding *ICD-10* codes.

For on-campus participants, multivariable piecewise exponential regression analysis was used to estimate the rate ratios (RRs) and 95% CIs for all-cause and cause-specific late mortality (overall health-related, subsequent neoplasm–related, and other health-related causes with a sufficient number of deaths to model) by modifiable CHCs and socioeconomic factors, adjusting for age at follow-up, sex, self-reported race and ethnicity (included based on previous reports suggesting racial and ethnic mortality differences in the general population), annual household income, and health insurance status.

All tests were 2-sided, with *P* < .05 considered statistically significant. Data were analyzed using SAS software, version 9.4 (SAS Institute Inc).

## Results

Characteristics and SMRs of 9440 individuals (8159 living and 1281 deceased) eligible to participate in the SJLIFE study and 3407 adult on-campus participants in the SJLIFE study are provided in Table 1 and eTable 2 in Supplement 1. Among these eligible survivors, the median (range) age at assessment was 27.5 (5.3-71.9) years, and the median (range) duration of follow-up was 18.8 (5.0-58.0) years. Most survivors were male (55.2%) and of non-Hispanic White race and ethnicity (75.3%). Similarly, among adult on-campus participants, the median (range) age at assessment was 35.4 (17.9-69.8) years, the median (range) duration of follow-up was 27.3 (7.3-54.7) years, and most were male (52.5%) and of non-Hispanic White race and ethnicity (81.7%). Specific causes of death are shown in eTable 3 in Supplement 1.

**Table 1.** Participant Characteristics and Standardized Mortality Ratios

Characteristic	All SJLIFE eligible survivors (n = 9440)^a^			Adult on-campus SJLIFE participants (n = 3407)^b^		
	No. (%)		SMR (95% CI)	No. (%)		SMR (95% CI)
	Alive	Dead		Alive	Dead	
Overall	8159	1281	7.6 (7.2-8.1)	3284	123	3.8 (3.1-4.5)
Race and ethnicity						
Hispanic	333 (4.1)	30 (2.3)	13.6 (9.2-19.4)	72 (2.2)	1 (0.8)	2.7 (0-15.2)
Non-Hispanic Black	1387 (17.0)	201 (15.7)	8.5 (7.4-9.8)	483 (14.7)	15 (12.2)	3.7 (2.1-6.1)
Non-Hispanic White	6151 (75.4)	955 (74.6)	6.9 (6.5-7.4)	2677 (81.5)	106 (86.2)	3.8 (3.1-4.6)
Other^c^	288 (3.5)	95 (7.4)	21.8 (17.6-26.6)	52 (1.6)	1 (0.8)	2.4 (0-13.4)
Sex						
Male	4437 (54.4)	770 (60.1)	6.3 (5.9-6.8)	1712 (52.1)	75 (61.0)	3.5 (2.8-4.4)
Female	3722 (45.6)	511 (39.9)	11.0 (10.1-12.0)	1572 (47.9)	48 (39.0)	4.3 (3.1-5.6)
Age at diagnosis, y						
0-4	3532 (43.3)	453 (35.4)	8.9 (8.1-9.7)	1217 (37.1)	33 (26.8)	3.4 (2.3-4.7)
5-9	1850 (22.7)	291 (22.7)	8.0 (7.1-9.0)	770 (23.4)	26 (21.1)	3.6 (2.3-5.2)
10-14	1587 (19.5)	292 (22.8)	7.0 (6.2-7.8)	761 (23.2)	31 (25.2)	3.9 (2.6-5.5)
15-19	1123 (13.8)	220 (17.2)	6.2 (5.4-7.1)	514 (15.7)	30 (24.4)	4.2 (2.8-6.0)
≥20	67 (0.8)	25 (2.0)	7.0 (4.5-10.3)	22 (0.7)	3 (2.4)	5.9 (1.2-17.1)
Decade of diagnosis						
1960-1969	139 (1.7)	126 (9.8)	6.6 (5.5-7.9)	85 (2.6)	13 (10.6)	3.9 (2.1-6.7)
1970-1979	804 (9.9)	352 (27.5)	6.4 (5.7-7.1)	525 (16.0)	48 (39.0)	4.6 (3.4-6.1)
1980-1989	1645 (20.2)	351 (27.4)	6.5 (5.8-7.2)	1042 (31.7)	40 (32.5)	3.6 (2.6-4.9)
1990-1999	2089 (25.6)	254 (19.8)	8.6 (7.6-9.7)	1210 (36.8)	18 (14.6)	2.6 (1.5-4.1)
2000-2012	3482 (42.7)	198 (15.5)	19.3 (16.7-22.2)	422 (12.9)	4 (3.3)	4.4 (1.2-11.3)
Age at assessment, y						
5-9	377 (4.6)	107 (8.4)	1047.4 (858.4-1265.7)	NA	NA	NA
10-14	778 (9.5)	218 (17.0)	377.7 (329.2-431.3)	NA	NA	NA
15-19	1055 (12.9)	208 (16.2)	87.7 (76.1-100.4)	22 (0.7)	0	0 (0-982.3)
20-24	1134 (13.9)	179 (14.0)	29.6 (25.4-34.2)	347 (10.6)	3 (2.4)	5.1 (1.0-14.8)
25-29	1110 (13.6)	141 (11.0)	11.5 (9.7-13.6)	566 (17.2)	9 (7.3)	3.6 (1.6-6.8)
30-39	2026 (24.8)	197 (15.4)	4.5 (3.9-5.2)	1284 (39.1)	34 (27.6)	3.8 (2.6-5.3)
40-49	1210 (14.8)	152 (11.9)	2.9 (2.4-3.3)	767 (23.4)	41 (33.3)	3.9 (2.8-5.3)
≥50	469 (5.7)	79 (6.2)	1.6 (1.3-2.0)	298 (9.1)	36 (29.3)	3.5 (2.5-4.9)
Survival after diagnosis, No. of y						
5-9	1572 (19.3)	539 (42.1)	24.8 (22.8-27.0)	27 (0.8)	0	0 (0-225.1)
10-14	1386 (17.0)	186 (14.5)	7.3 (6.3-8.5)	209 (6.4)	4 (3.3)	3.3 (0.9-8.6)
15-19	1205 (14.8)	137 (10.7)	5.1 (4.3-6.1)	508 (15.5)	5 (4.1)	2.1 (0.9-4.4)
20-24	1064 (13.0)	109 (8.5)	4.3 (3.6-5.2)	670 (20.4)	19 (15.4)	4.0 (2.3-6.3)
25-29	857 (10.5)	84 (6.6)	3.9 (3.1-4.8)	542 (16.5)	18 (14.6)	3.2 (1.9-5.1)
30-34	836 (10.2)	83 (6.5)	4.2 (3.3-5.2)	537 (16.4)	18 (14.6)	3.0 (1.8-4.7)
35-39	650 (8.0)	82 (6.4)	5.8 (4.6-7.2)	413 (12.6)	31 (25.2)	5.8 (3.9-8.1)
≥40	589 (7.2)	61 (4.8)	4.5 (3.4-5.7)	378 (11.5)	28 (22.8)	4.0 (2.6-5.9)
Cancer diagnosis						
Leukemia						
Acute lymphoblastic	2212 (27.1)	303 (23.7)	6.2 (5.5-6.9)	1054 (32.1)	25 (20.3)	2.2 (1.4-3.2)
Acute myeloid	379 (4.6)	38 (3.0)	7.9 (5.6-10.8)	125 (3.8)	4 (3.3)	4.5 (1.2-11.6)
Chronic myeloid	13 (0.2)	2 (0.2)	13.8 (1.5-49.8)	3 (0.1)	0	15.9 (1.8-57.3)
Other	61 (0.7)	14 (1.1)	13.3 (7.3-22.3)	22 (0.7)	2 (1.6)	0 (0-242.9)
Central nervous system tumor						
Astrocytoma	618 (7.6)	86 (6.7)	15.5 (12.4-19.2)	181 (5.5)	4 (3.3)	4.3 (1.2-11.1)
Medulloblastoma	341 (4.2)	54 (4.2)	17.8 (13.3-23.2)	92 (2.8)	3 (2.4)	5.1 (1.0-15.0)
Ependymoma	188 (2.3)	64 (5.0)	60.3 (46.4-77.0)	37 (1.1)	4 (3.3)	18.9 (5.1-48.5)
Other	165 (2.0)	20 (1.6)	14.6 (8.9-22.6)	32 (1.0)	0	0 (0-21.1)
Craniopharyngioma	141 (1.7)	13 (1.0)	18.4 (9.8-31.5)	31 (0.9)	0	0 (0-26.3)
Hodgkin lymphoma	682 (8.4)	185 (14.4)	7.2 (6.2-8.4)	363 (11.1)	45 (36.6)	8.4 (6.1-11.3)
Non-Hodgkin lymphoma	503 (6.2)	74 (5.8)	4.6 (3.6-5.8)	236 (7.2)	8 (6.5)	3.2 (1.4-6.2)
Kidney	473 (5.8)	50 (3.9)	5.4 (4.0-7.2)	214 (6.5)	6 (4.9)	3.5 (1.3-7.6)
Neuroblastoma	379 (4.6)	73 (5.7)	12.0 (9.4-15.1)	149 (4.5)	1 (0.8)	0.9 **(**0-5.0)
Soft tissue sarcoma	492 (6.0)	77 (6.0)	6.9 (5.5-8.6)	210 (6.4)	5 (4.1)	2.8 (0.9-6.6)
Ewing sarcoma	198 (2.4)	66 (5.2)	10.1 (7.8-12.8)	100 (3.0)	2 (1.6)	1.5 (0.2-5.4)
Osteosarcoma	237 (2.9)	52 (4.1)	5.9 (4.4-7.7)	126 (3.8)	4 (3.3)	2.0 (0.5-5.1)
Retinoblastoma	428 (5.2)	30 (2.3)	6.6 (4.4-9.4)	96 (2.9)	3 (2.4)	3.4 (0.7-10.0)
Germ cell tumor	179 (2.2)	18 (1.4)	5.0 (3.0-7.9)	81 (2.5)	2 (1.6)	3.6 (0.4-13.1)
Liver cancers	65 (0.8)	5 (0.4)	4.7 (1.5-11.0)	22 (0.7)	1 (0.8)	7.8 (0.1-43.4)
Melanoma	71 (0.9)	7 (0.5)	5.8 (2.3-12.0)	21 (0.6)	0	0 (0-17.6)
Nasopharyngeal carcinoma	46 (0.6)	15 (1.2)	9.4 (5.2-15.4)	22 (0.7)	4 (3.3)	11.7 (3.2-30.0)
Histiocytosis	138 (1.7)	7 (0.5)	2.0 (0.8-4.1)	21 (0.6)	0	0 (0-22.2)
Other cancers	150 (1.8)	28 (2.2)	12.1 (8.0-17.5)	46 (1.4)	0	0 (0-10.0)
**Cancer treatment**						
Radiotherapy						
Total body						
No	7898 (96.8)	1222 (95.4)	7.4 (7.0-7.8)	3188 (97.1)	121 (98.4)	3.8 (3.1-4.5)
Yes	261 (3.2)	39 (3.0)	13.1 (9.3-17.9)	96 (2.9)	2 (1.6)	3.9 (0.4-14.1)
Cranial						
No	5849 (71.7)	689 (53.8)	6.2 (5.7-6.7)	2267 (69.0)	78 (63.4)	3.8 (3.0-4.7)
Yes	2310 (28.3)	577 (45.0)	10.2 (9.4-11.1)	1017 (31.0)	45 (36.6)	3.8 (2.7-5.0)
Craniospinal						
No	7793 (95.5)	1091 (85.2)	6.7 (6.3-7.1)	3175 (96.7)	116 (94.3)	3.7 (3.0-4.4)
Yes	365 (4.5)	88 (6.9)	16.3 (13.1-20.1)	109 (3.3)	7 (5.7)	6.5 (2.6-13.3)
Chest						
No	6418 (78.7)	712 (55.6)	5.9 (5.4-6.3)	2528 (77.0)	56 (45.5)	2.4 (1.8-3.1)
Yes	1741 (21.3)	551 (43.0)	11.9 (10.9-12.9)	756 (23.0)	67 (54.5)	7.5 (5.8-9.5)
Abdominal or pelvic						
No	6420 (78.7)	716 (55.9)	6.0 (5.6-6.5)	2528 (77.0)	58 (47.2)	2.4 (1.9-3.2)
Yes	1739 (21.3)	547 (42.7)	11.2 (10.3-12.2)	756 (23.0)	65 (52.8)	7.2 (5.6-9.2)
Other site(s)						
No	7802 (95.6)	1136 (88.7)	7.2 (6.8-7.6)	3127 (95.2)	119 (96.7)	3.8 (3.2-4.6)
Yes	356 (4.4)	38 (3.0)	4.0 (2.8-5.4)	157 (4.8)	4 (3.3)	2.3 (0.6-5.9)
Chemotherapy						
Alkylators						
No	3620 (44.4)	413 (32.2)	6.6 (6.0-7.3)	1368 (41.7)	38 (30.9)	3.3 (2.3-4.6)
Yes	4539 (55.6)	847 (66.1)	8.0 (7.5-8.6)	1916 (58.3)	85 (69.1)	4.0 (3.2-4.9)
Anthracyclines						
No	3827 (46.9)	704 (55.0)	7.9 (7.3-8.5)	1365 (41.6)	75 (61.0)	4.8 (3.7-6.0)
Yes	4332 (53.1)	557 (43.5)	7.1 (6.5-7.7)	1919 (58.4)	48 (39.0)	2.8 (2.1-3.8)
Platinum						
No	6694 (82.0)	1001 (78.1)	6.5 (6.1-6.9)	2868 (87.3)	108 (87.8)	3.6 (2.9-4.3)
Yes	1465 (18.0)	260 (20.3)	19.9 (17.6-22.5)	416 (12.7)	15 (12.2)	5.9 (3.3-9.7)
Anthracycline dose, mg/m^2^						
None	3827 (46.9)	704 (55.0)	7.9 (7.3-8.5)	1365 (41.6)	75 (61.0)	4.8 (3.7-6.0)
0.1 to <250.0	3383 (41.5)	320 (25.0)	5.9 (5.2-6.5)	1481 (45.1)	35 (28.5)	3.0 (2.1-4.1)
≥250.0	892 (10.9)	203 (15.8)	8.8 (7.7-10.1)	433 (13.2)	13 (10.6)	2.6 (1.4-4.4)
ADI, %						
1-10^d^^,^^e^	NA	NA	NA	326 (9.9)	2 (1.6)	0.6 (0.1-2.2)
11-50	NA	NA	NA	1278 (38.9)	30 (24.4)	2.4 (1.6-3.4)
51-80	NA	NA	NA	942 (28.7)	40 (32.5)	4.1 (2.9-5.6)
81-100	NA	NA	NA	590 (18.0)	32 (26.0)	5.7 (3.9-8.0)
Unassigned	NA	NA	NA	148 (4.5)	19 (15.4)	12.7 (7.6-19.8)
Annual household income, $^e^^,^^f^						
<20 000	NA	NA	NA	483 (14.7)	19 (15.4)	4.3 (2.6-6.8)
20 000-59 000	NA	NA	NA	1023 (31.2)	47 (38.2)	4.5 (3.3-6.0)
60 000-99 000	NA	NA	NA	662 (20.2)	22 (17.9)	3.1 (1.9-4.6)
≥100 000	NA	NA	NA	566 (17.2)	15 (12.2)	2.1 (1.2-3.5)
Missing	NA	NA	NA	550 (16.7)	20 (16.3)	5.4 (3.3-8.3)
Health insurance status^e^						
None	NA	NA	NA	698 (21.3)	25 (20.3)	3.7 (2.4-5.4)
Public	NA	NA	NA	542 (16.5)	47 (38.2)	9.2 (6.8-12.3)
Private	NA	NA	NA	1949 (59.3)	46 (37.4)	2.3 (1.7-3.0)
Missing	NA	NA	NA	95 (2.9)	5 (4.1)	7.4 (2.4-17.2)

Abbreviations: ADI, area deprivation index; NA, not applicable; SJLIFE, St Jude Lifetime Cohort; SMR, standardized mortality ratio.

^a^ Follow-up began 5 years after cancer diagnosis.

^b^ Follow-up began after the baseline on-campus assessment. Limited to adult (aged ≥18 years) SJLIFE participants with an on-campus visit before December 31, 2016.

^c^ Other race and ethnicity category includes 33 Black and 139 White participants with unknown Hispanic ethnicity status, 13 participants with unknown race and unknown Hispanic ethnicity status, 8 participants of other races with unknown Hispanic ethnicity status, and 190 participants of other races with non-Hispanic ethnicity (8 American Indian or Alaska Native, 41 Asian, 14 Asian and White, 26 Black and White, 11 multiple races [not specified], 5 Pacific Islander, 78 other races [not specified], and 7 missing).

^d^ Reference category.

^e^ Data were not available for nonparticipants.

^f^ Income was adjusted to 2016 dollars using inflation rate of 3% per year.

**Figure 1.**
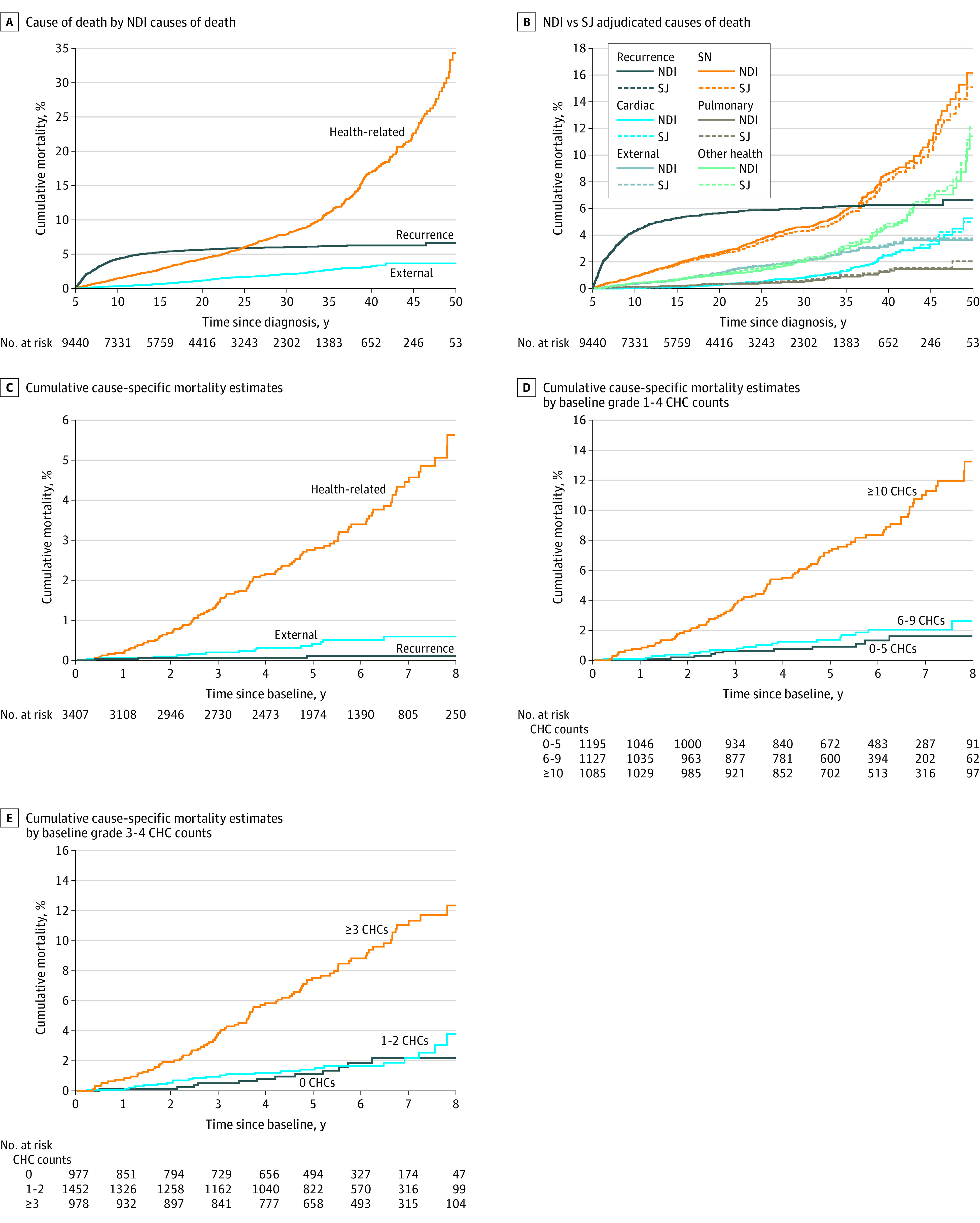
Cumulative Cause-Specific Mortality Estimates Among 5-Year Survivors of Childhood Cancer Data were based on 168 chronic health conditions (CHCs) routinely graded at baseline as part of the St Jude Lifetime Cohort assessment of adult survivors of childhood cancer. NDI indicates National Death Index; SJ, St Jude Children’s Research Hospital; and SN, subsequent neoplasm.

Mortality rates by cause of death and time from cancer diagnosis are shown in eTable 4 in Supplement 1. Between 5 and 9 years after cancer diagnosis, the highest rate of death was due to recurrent cancer (9.2 deaths/1000 person-years; 95% CI, 8.3-10.1 deaths/1000 person-years), whereas rates of health-related causes of death exceeded that of recurrence beginning at 10 to 14 years after diagnosis and beyond (ranging from 2.8 deaths/1000 person-years at 10-14 years after diagnosis to 19.4 deaths/1000 person-years at ≥40 years after diagnosis). The rate of subsequent neoplasm–related deaths ranged from 1.9 per 1000 person-years (5-9 years after diagnosis) to 2.3 per 1000 person-years (20-24 years after diagnosis) between 5 and 29 years after diagnosis but increased to 8.8 per 1000 person-years by 40 or more years after diagnosis. The cumulative incidence of health-related causes of death exceeded cancer recurrence or progression by 25 years after diagnosis (Figure 1A). Cumulative cause-specific mortality estimates beginning at 5 years after cancer diagnosis are shown in Figure 1B, and cumulative cause-specific mortality estimates beginning at baseline SJLIFE assessment are shown in Figure 1C.

Childhood cancer survivors experienced a 7.6-fold increased rate of both all-cause mortality (SMR, 7.6; 95% CI, 7.2-8.1) and health-related mortality (SMR, 7.6; 95% CI, 7.0-8.2) (eTable 5 in Supplement 1). Among specific health-related causes of death, SMRs were 16.0 (95% CI, 14.4-17.8) for subsequent neoplasms, 4.2 (95% CI, 3.3-5.3) for cardiac causes, 9.0 (95% CI, 6.5-12.0) for pulmonary causes, and 4.3 (95% CI, 3.7-5.0) for other health-related causes.

### Modifiable Risk Factors

Among on-campus participants, adjusting for age during follow-up, age at diagnosis, sex, race and ethnicity, treatment exposures, annual household income, insurance status, and healthy lifestyle index score, having 1 modifiable CHC of grade 2 or higher (RR, 2.2; 95% CI, 1.2-4.0; *P* = .01), 2 modifiable CHCs of grade 2 or higher (RR, 2.6; 95% CI, 1.4-4.9; *P* = .003), or 3 modifiable CHCs of grade 2 or higher (RR, 3.6; 95% CI, 1.8-7.1; *P* < .001); living in a Census block with an ADI in the 51st to 80th percentile (RR, 5.5; 95% CI, 1.3-23.5; *P* = .02), an ADI in the 81st to 100th percentile (RR, 8.7; 95% CI, 2.0-37.6; *P* = .004), or an unassigned ADI (RR, 15.7; 95% CI, 3.5-70.3; *P* < .001); and having frailty (RR, 2.3; 95% CI, 1.3-3.9; *P* = .004) were associated with an increased risk of subsequent all-cause death. In addition, having 1 modifiable CHC of grade 2 or higher (RR, 2.2; 95% CI, 1.1-4.4; *P* = .02), 2 modifiable CHCs of grade 2 or higher (RR, 2.5; 95% CI, 1.2-5.2; *P* = .01), or 3 modifiable CHCs of grade 2 or higher (RR, 4.0; 95% CI, 1.9-8.4; *P* < .001); living in a Census block with an ADI in the 51st to 80th percentile (RR, 9.2; 95% CI, 1.2-69.7; *P* = .03), an ADI in the 81st to 100th percentile (RR, 16.2, 95% CI, 2.1-123.7; *P* = .007), or an unassigned ADI (RR, 27.3; 95% CI, 3.5-213.6; *P* = .002); and having frailty (RR, 2.3; 95% CI, 1.2-4.1; *P* = .009) were associated with an increased risk of subsequent health-related death. Prefrailty was also associated with an increased risk of health-related death (RR, 1.7; 95% CI, 1.0-2.7; *P* = .049) (Table 2).

**Table 2.** Multivariable Associations Between Modifiable Risk Factors and Postbaseline All-Cause and Cause-Specific Mortality Rates Among On-Campus Adult Participants in the St Jude Lifetime Cohort

Variable	Death by cause (n = 3407)^a^											
	All-cause		Health-related								External	
	RR (95% CI)	*P* value	All health-related		Health-related excluding subsequent neoplasm		Subsequent neoplasm		Other health-related		RR (95% CI)	*P* value
			RR (95% CI)	*P* value	RR (95% CI)	*P* value	RR (95% CI)	*P* value	RR (95% CI)	*P* value		
Age at diagnosis, y												
<5	1 [Reference]	NA	1 [Reference]	NA	1 [Reference]	NA	1 [Reference]	NA	1 [Reference]	NA	1 [Reference]	NA
5-9	0.8 (0.5-1.4)	.44	0.8 (0.4-1.5)	.47	0.7 (0.3-1.7)	.50	0.9 (0.3-2.2)	.76	1.0 (0.4-2.6)	.93	0.9 (0.2-5.2)	.95
10-14	0.8 (0.4-1.3)	.34	0.7 (0.4-1.3)	.25	0.6 (0.2-1.4)	.22	1.0 (0.4-2.5)	.93	0.2 (0.1-0.9)	.04	1.4 (0.3-7.3)	.69
15-19	0.8 (0.4-1.5)	.50	0.8 (0.4-1.5)	.40	0.7 (0.3-1.8)	.47	0.9 (0.3-2.5)	.90	0.8 (0.3-2.4)	.72	1.8 (0.3-10.5)	.51
Sex												
Female	1 [Reference]	NA	1 [Reference]	NA	1 [Reference]	NA	1 [Reference]	NA	1 [Reference]	NA	1 [Reference]	NA
Male	1.9 (1.3-2.9)	.002	1.8 (1.2-2.8)	.01	2.6 (1.4-4.8)	.003	1.2 (0.6-2.3)	.57	1.8 (0.9-3.9)	.12	5.0 (1.2-20.2)	.02
Race												
White	1 [Reference]	NA	1 [Reference]	NA	1 [Reference]	NA	1 [Reference]	NA	1 [Reference]	NA	1 [Reference]	NA
Non-White	0.8 (0.5-1.4)	.46	0.9 (0.5-1.7)	.81	1.0 (0.5-2.3)	.96	0.8 (0.3-1.9)	.55	1.7 (0.7-4.5)	.27	0.6 (0.1-3.3)	.56
Annual household income, $												
<20 000	0.7 (0.3-1.6)	.39	0.8 (0.3-1.9)	.55	0.5 (0.2-1.8)	.31	1.3 (0.3-5.1)	.72	0.4 (0.1-1.8)	.21	0.1 (0-2.1)	.16
20 000-59 000	1.1 (0.5-2.1)	.87	1.1 (0.5-2.3)	.77	0.7 (0.3-1.9)	.48	2.1 (0.7-6.8)	.21	0.6 (0.2-2.2)	.47	0.7 (0.1-4.3)	.68
60 000-99 000	0.9 (0.4-1.8)	.78	1.0 (0.5-2.1)	.93	0.6 (0.2-1.7)	.38	1.8 (0.5-6.2)	.35	0.6 (0.2-2.3)	.48	0.5 (0.1-4.3)	.56
≥100 000	1 [Reference]	NA	1 [Reference]	NA	1 [Reference]	NA	1 [Reference]	NA	1 [Reference]	NA	1 [Reference]	NA
Missing	1.3 (0.6-3.0)	.54	1.3 (0.5-3.4)	.55	1.9 (0.6-6.2)	.27	0.6 (0.1-3.9)	.63	2.0 (0.5-8.3)	.35	0.4 (0-4.2)	.47
Health insurance status												
None	1.3 (0.7-2.3)	.37	1.1 (0.6-2.1)	.72	1.0 (0.4-2.3)	.93	1.4 (0.5-3.5)	.52	0.6 (0.2-2.0)	.43	3.2 (0.7-14.2)	.13
Public	2.8 (1.6-4.6)	<.001	2.4 (1.4-4.2)	.002	2.5 (1.2-5.6)	.02	2.5 (1.1-5.5)	.03	2.3 (0.9-6.1)	.08	4.7 (0.9-23.3)	.06
Private	1 [Reference]	NA	1 [Reference]	NA	1 [Reference]	NA	1 [Reference]	NA	1 [Reference]	NA	1 [Reference]	NA
Missing	1.7 (0.5-6.3)	.42	1.3 (0.3-6.1)	.74	1.4 (0.3-7.4)	.68	0 (0-8.2)	.45	2.4 (0.4-13.9)	.34	9.0 (0.6-137.3)	.12
Grade ≥2 modifiable CHCs^b^^,^^c^												
None	1 [Reference]	NA	1 [Reference]	NA	1 [Reference]	NA	1 [Reference]	NA	1 [Reference]	NA	1 [Reference]	NA
1	2.2 (1.2-4.0)	.01	2.2 (1.1-4.4)	.02	2.2 (0.8-5.6)	.11	2.5 (0.9-6.9)	.07	6.1 (1.3-28.4)	.02	2.7 (0.7-10.7)	.17
2	2.6 (1.4-4.9)	.003	2.5 (1.2-5.2)	.01	3.0 (1.1-7.9)	.03	2.2 (0.7-6.5)	.16	5.9 (1.2-29.4)	.03	2.4 (0.4-13.3)	.32
3	3.6 (1.8-7.1)	<.001	4.0 (1.9-8.4)	<.001	5.1 (1.9-13.7)	.001	3.2 (1.0-10.2)	.04	12.9 (2.6-63.6)	.002	1.7 (0.2-16.9)	.66
≥4	1.8 (0.7-4.5)	.19	1.8 (0.7-4.8)	.25	1.3 (0.3-5.7)	.69	2.3 (0.6-9.2)	.23	4.4 (0.7-29.2)	.12	2.7 (0.2-35.1)	.45
Healthy lifestyle index^d^												
0 (Unhealthy in all 4 domains)	2.7 (0.6-12.5)	.20	4.1 (0.5-33.0)	.19	2.3 (0.3-20.8)	.45	2.1 (0.2-19.9)	.53	3.4 (0.4-32.2)	.28	0.3 (0-7.8)	.50
1	1.8 (0.4-7.8)	.42	2.8 (0.4-21.2)	.32	1.2 (0.1-10.0)	.87	2.0 (0.2-15.9)	.52	0.9 (0.1-8.5)	.94	1.0 (0.1-10.0)	.99
2	1.8 (0.4-7.6)	.43	3.0 (0.4-22.2)	.28	1.9 (0.2-15.2)	.53	1.1 (0.1-8.7)	.93	1.3 (0.2-10.7)	.81	0.5 (0-4.7)	.51
3	2.0 (0.5-8.7)	.34	3.5 (0.5-26.2)	.22	1.5 (0.2-12.1)	.72	2.1 (0.3-16.1)	.49	1.2 (0.1-10.6)	.86	0.5 (0-5.6)	.57
4 (Healthy in all 4 domains)	1 [Reference]	NA	1 [Reference]	NA	1 [Reference]	NA	1 [Reference]	NA	1 [Reference]	NA	1 [Reference]	NA
ADI, %												
1-10	1 [Reference]	NA	1 [Reference]	NA	NA	NA	1 [Reference]	NA	NA	NA	1 [Reference]	NA
11-50	3.7 (0.9-16.0)	.08	7.2 (1.0-54.4)	.05	1 [Reference]^e^	NA	2.7 (0.4-21.4)	.34	1 [Reference]^e^	NA	0.5 (0-5.9)	.58
51-80	5.5 (1.3-23.5)	.02	9.2 (1.2-69.7)	.03	1.4 (0.6-3.1)	.40	3.1 (0.4-24.7)	.28	0.9 (0.3-2.4)	.82	2.1 (0.2-19.6)	.53
81-100	8.7 (2.0-37.6)	.004	16.2 (2.1-123.7)	.007	3.1 (1.4-7.0)	.007	4.8 (0.6-39.4)	.14	3.1 (1.2-8.2)	.02	2.1 (0.2-23.0)	.53
Unassigned	15.7 (3.5-70.3)	<.001	27.3 (3.5-213.6)	.002	4.7 (1.8-11.9)	.001	9.6 (1.1-82.9)	.04	1.5 (0.4-6.4)	.55	2.8 (0.1-56.5)	.50
Frailty												
No frailty	1 [Reference]	NA	1 [Reference]	NA	1 [Reference]	NA	1 [Reference]	NA	1 [Reference]	NA	1 [Reference]	NA
Prefrailty	1.4 (0.9-2.3)	.13	1.7 (1.0-2.7)	.049	1.5 (0.7-3.1)	.28	2.0 (1.0-4.1)	.06	1.5 (0.6-3.8)	.40	0.8 (0.2-4.3)	.83
Frailty	2.3 (1.3-3.9)	.004	2.3 (1.2-4.1)	.009	3.3 (1.5-7.4)	.003	1.5 (0.6-4.1)	.40	2.8 (1.0-8.2)	.05	4.1 (1.0-16.7)	.05
**Treatment**												
Alkylator dose, mg/m^2^												
None	1 [Reference]	NA	1 [Reference]	NA	1 [Reference]	NA	1 [Reference]	NA	1 [Reference]	NA	1 [Reference]	NA
<8000	1.0 (0.6-1.8)	.99	0.9 (0.5-1.7)	.71	0.6 (0.2-1.5)	.24	1.5 (0.6-3.9)	.38	0.3 (0.1-1.2)	.09	1.9 (0.5-8.2)	.37
≥8000	1.0 (0.6-1.6)	.92	1.0 (0.6-1.7)	.96	0.8 (0.4-1.7)	.65	1.2 (0.5-2.8)	.61	0.8 (0.3-1.9)	.63	0.4 (0.1-2.7)	.38
Anthracycline dose, mg/m^2^												
None	1 [Reference]	NA	1 [Reference]	NA	1 [Reference]	NA	1 [Reference]	NA	1 [Reference]	NA	1 [Reference]	NA
0.1 to <250.0	0.8 (0.5-1.3)	.41	0.7 (0.4-1.1)	.13	0.6 (0.3-1.3)	.17	0.7 (0.3-1.4)	.30	0.9 (0.3-2.2)	.76	1.5 (0.3-7.2)	.64
≥250.0	0.7 (0.3-1.4)	.30	0.8 (0.4-1.7)	.56	1.2 (0.5-2.9)	.61	0.3 (0.1-1.3)	.10	1.0 (0.3-3.8)	.97	0.4 (0-4.3)	.43
Cranial radiotherapy dose, Gy												
None	1 [Reference]	NA	1 [Reference]	NA	1 [Reference]	NA	1 [Reference]	NA	1 [Reference]	NA	1 [Reference]	NA
<20	0.6 (0.2-1.6)	.29	0.9 (0.3-2.5)	.88	0.5 (0.1-2.7)	.45	1.4 (0.4-4.8)	.63	1.4 (0.3-7.8)	.69	0 (0-0.6)	.02
20 to <30	0.7 (0.4-1.3)	.23	0.9 (0.5-1.7)	.77	0.8 (0.3-1.9)	.61	1.2 (0.5-2.9)	.71	1.6 (0.6-4.4)	.33	0 (0-0.7)	.03
≥30	0.8 (0.4-1.5)	.49	0.9 (0.4-1.8)	.71	0.3 (0.1-1.0)	.047	2.2 (0.9-5.7)	.10	0.9 (0.2-3.2)	.81	1.0 (0.2-6.6)	.99
Chest radiotherapy dose, Gy												
None	1 [Reference]	NA	1 [Reference]	NA	1 [Reference]	NA	1 [Reference]	NA	1 [Reference]	NA	1 [Reference]	NA
<20	2.7 (1.2-6.1)	.02	2.9 (1.2-7.2)	.02	7.6 (2.4-23.8)	<.001	0.9 (0.2-4.4)	.88	6.8 (1.8-25.3)	.005	2.5 (0.3-24.0)	.43
≥20	3.2 (2.1-5.0)	<.001	3.9 (2.4-6.3)	<.001	6.9 (3.5-13.7)	<.001	2.1 (1.0-4.3)	.047	5.5 (2.3-13.0)	<.001	1.0 (0.3-3.9)	.97

Abbreviations: ADI, area deprivation index; CHC, chronic health condition; NA, not applicable; RR, rate ratio.

^a^ Adjusted for age during follow-up as cubic splines.

^b^ Excluding underweight and obesity due to inclusion in the healthy lifestyle index.

^c^ Including dyslipidemia, hypertension, diabetes, bone mineral deficiency, hypogonadism, hypothyroidism, and adrenal insufficiency.

^d^ Domains included smoking status, alcohol consumption, physical activity, and body mass index (calculated as weight in kilograms divided by height in meters squared).

Adjusting for the same variables, we explored the association between the presence of specific modifiable CHCs at baseline assessment and subsequent risk of death (eTable 6 in Supplement 1). Having grade 2 hypertension (RR, 2.1; 95% CI, 1.2-3.7; *P* = .01), grade 3 hypertension (RR, 2.2; 95% CI, 1.1-4.3; *P* = .03), and grade 3 diabetes (RR, 2.4; 95% CI, 1.1-5.3; *P* = .03) was associated with an increased risk of all-cause death, while having grade 2 dyslipidemia (RR, 0.6; 95% CI, 0.3-1.0; *P* = .04) was associated with a decreased risk of all-cause death. The presence of grade 2 hypertension (RR, 2.1; 95% CI, 1.1-3.9; *P* = .02) and grade 3 diabetes (RR, 3.2; 95% CI, 1.4-7.2; *P* = .005) at baseline was associated with an increased risk of health-related death, while the presence of grade 1 dyslipidemia (RR, 0.4; 95% CI, 0.2-0.9; *P* = .03) and grade 2 dyslipidemia (RR, 0.5; 95% CI, 0.3-0.8; *P* = .01) was associated with a decreased risk of health-related death. Similar associations were observed when excluding subsequent neoplasms from health-related causes. Having grade 3 diabetes (RR, 3.9; 95% CI, 1.3-11.7; *P* = .01) and grade 1 hypogonadism (RR, 2.7; 95% CI, 1.1-6.8; *P* = .04) was associated with an increased risk of subsequent neoplasm–related death, while having grade 3 diabetes (RR, 4.5; 95% CI, 1.1-17.6; *P* = .03) was associated with an increased risk of other health-related causes of death, and having grade 2 dyslipidemia (RR, 0.2; 95% CI, 0.1-0.7; *P* = .008) was associated with a decreased risk of other health-related causes of death.

### CHC Burden and Subsequent Mortality

**Figure 2.**
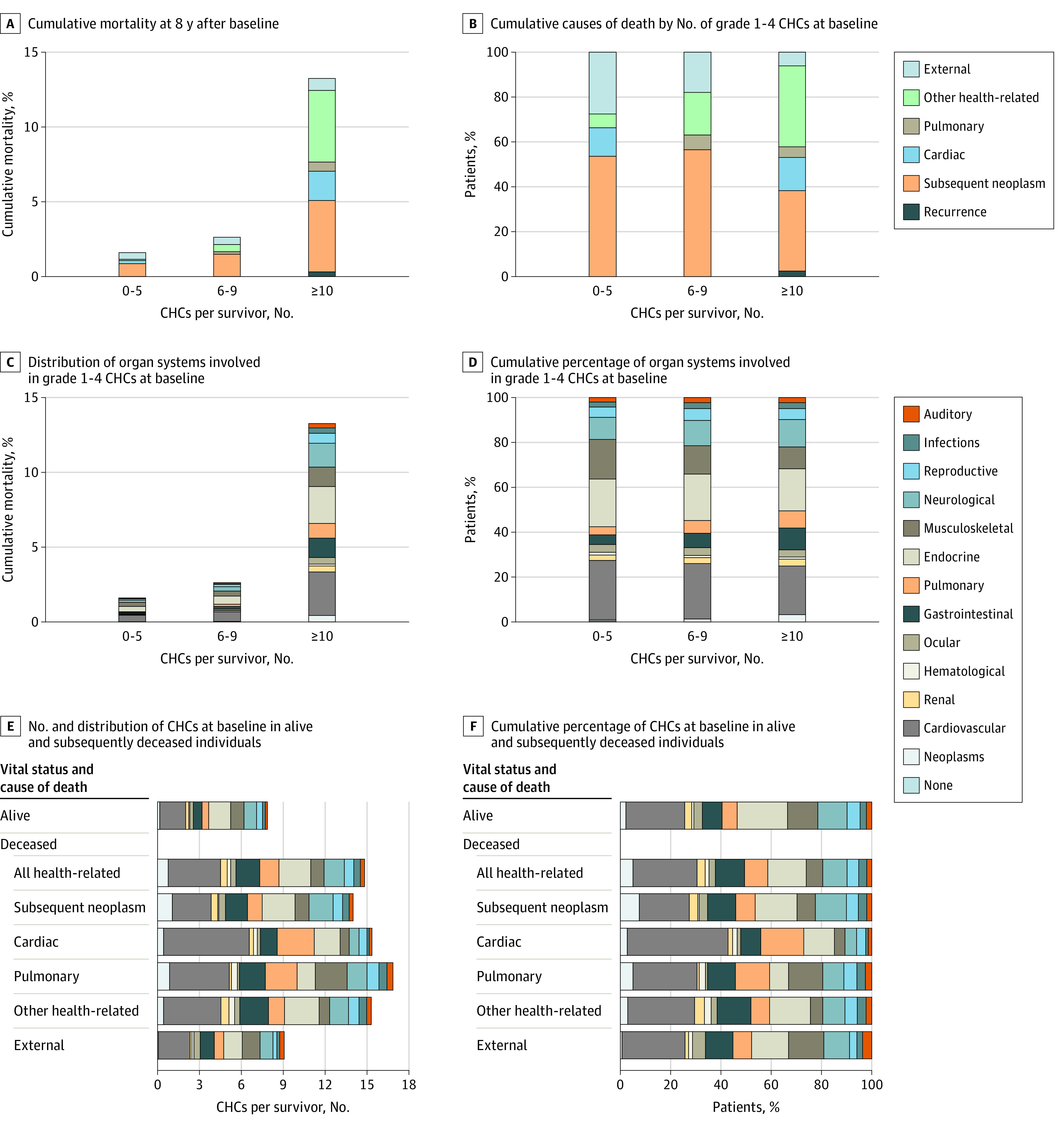
Cumulative Mortality and Cumulative Percentages of Causes of Death, Organ Systems Involved, and Chronic Health Conditions (CHCs) Among Participants With Grade 1 to 4 CHCs at Baseline Data were based on 168 CHCs routinely graded at baseline as part of the St Jude Lifetime Cohort assessment of adult survivors of childhood cancer.

**Figure 3.**
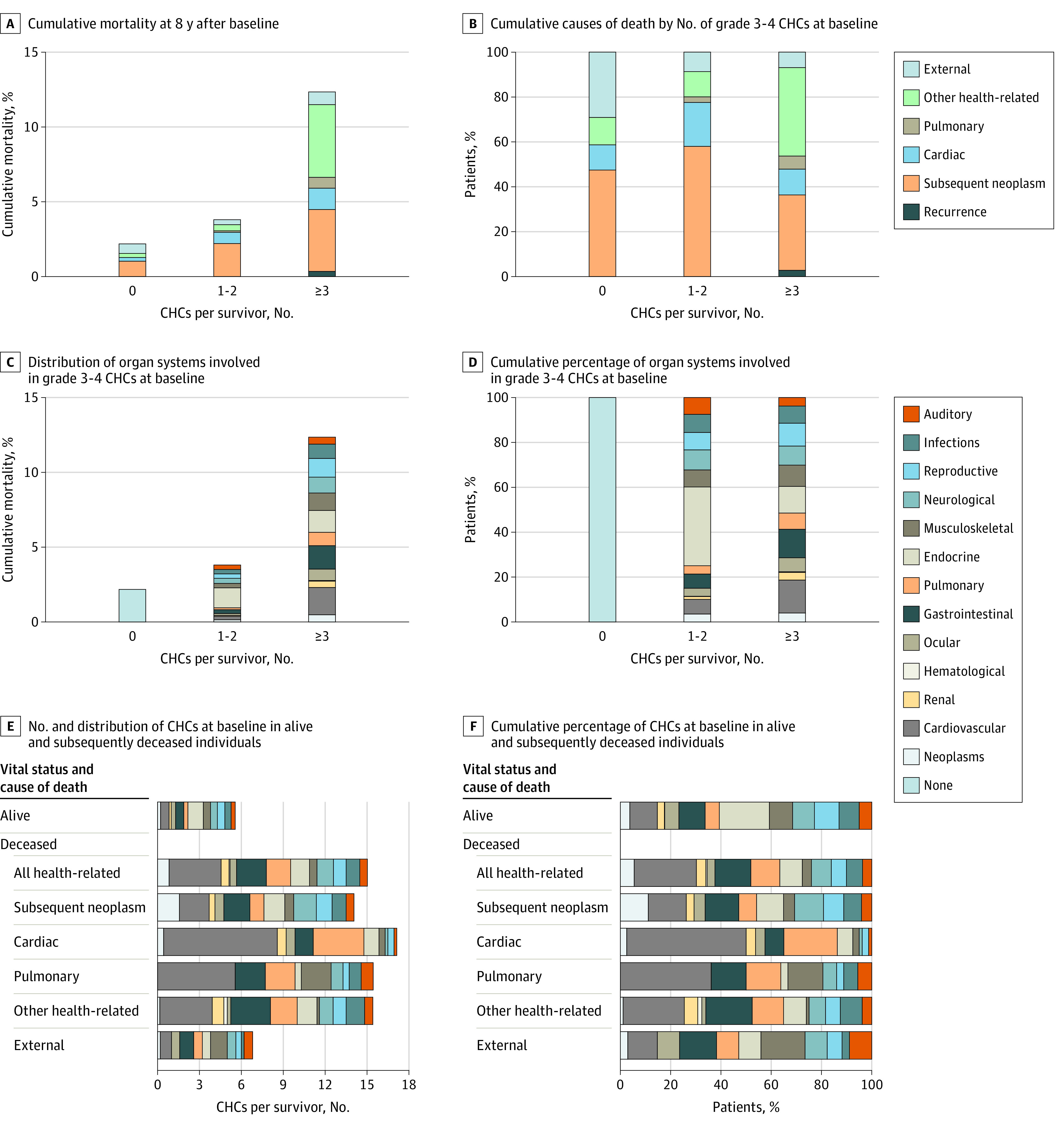
Cumulative Mortality and Cumulative Percentages of Causes of Death, Organ Systems Involved, and Chronic Health Conditions (CHCs) Among Participants With Grade 3 to 4 CHCs at Baseline Data were based on 168 CHCs routinely graded at baseline as part of the St Jude Lifetime Cohort assessment of adult survivors of childhood cancer.

Among 168 routinely graded (vs only modifiable) conditions, there was a significant increase in the risk of late death among individuals with 10 or more CHCs of grade 1 to 4 (Figure 1D) and among individuals with 3 or more CHCs of grade 3 to 4 at baseline (Figure 1E). The risk of late death in survivors with 6 to 9 CHCs of grade 1 to 4 was similar to that of individuals with 0 to 5 CHCs of grade 1 to 4, and the risk of late death in survivors with 1 to 2 CHCs of grade 3 to 4 at baseline was similar to that of individuals with 0 CHCs of grade 3 to 4 at baseline. Cumulative mortality 9 years after baseline assessment, including the respective distribution of causes of death, is shown by the number of grade 1 to 4 CHCs and grade 3 to 4 CHCs in Figure 2A and Figure 3A, respectively. Cumulative mortality 8 years after baseline assessment was 13.2% (95% CI, 10.2%-16.3%) in survivors with 10 or more CHCs of grade 1 to 4 at baseline compared with 1.6% (95% CI, 0.6%-2.6%) in those with 0 to 5 CHCs of grade 1 to 4 at baseline and 2.6% (95% CI, 1.1%-4.1%) in those with 6 to 9 CHCs of grade 1 to 4 at baseline. The cumulative percentages of each cause of death by the number of grade 1 to 4 CHCs and grade 3 to 4 CHCs are shown in Figure 2B and Figure 3B, respectively; the proportion of health-related causes of death increased with the number of CHCs identified at baseline.

The cumulative risk of late death by the number of baseline grade 1 to 4 CHCs and grade 3 to 4 CHCs, stratified by organ system, is shown in Figure 2C and Figure 3C, respectively. We observed similar distributions of organ systems involved, independent of the number of CHCs at baseline (proportions are shown in Figure 2D and Figure 3D).

The number and distribution (by organ system) of baseline grade 1 to 4 CHCs and grade 3 to 4 CHCs, respectively, among living and deceased survivors by cause of death are shown in Figure 2E and Figure 3E. The cumulative percentages (by organ system involvement) of baseline grade 1 to 4 CHCs and grade 3 to 4 CHCs, respectively, among living and deceased survivors by cause of death are shown in Figure 2F and Figure 3F. Deceased survivors, regardless of cause of death (except those who died of external causes), had more than 2.5 times the number of baseline grade 3 to 4 CHCs compared with those alive at last follow-up. In general, deceased survivors had more cardiovascular and pulmonary CHCs than those who were alive.

### Cause of Death Adjudication

After manual record review of the 1281 causes of death, we identified 99 discrepancies between NDI-assigned and SJLIFE-assigned causes (eTable 7 in Supplement 1). The most common discrepancies involved modification of NDI-assigned cancer-related causes for greater specificity or reclassification to specific noncancer conditions. There were no appreciable differences in cumulative mortality estimates using NDI-assigned compared with SJLIFE-assigned causes of death (Figure 1B).

## Discussion

In this longitudinal cohort study of data from the SJLIFE study, which involved a prospectively followed-up clinically assessed cohort, we identified robust associations of modifiable CHCs and socioeconomic factors with increased risk of late death in adult survivors of childhood cancer. We observed an approximately 2-fold or higher increased risk of death among survivors with 1 or more clinically assessed and validated modifiable CHCs despite adjustment for individual- and neighborhood-level sociodemographic factors, suggesting that the increased late mortality experienced by childhood cancer survivors in adulthood may not be predetermined by treatment-related risk factors alone.

While modifiable CHCs (eg, hypertension) have been found to increase the incidence of specific outcomes (eg, heart failure) in survivors,^7^ their associations with late all-cause mortality were previously unknown. Survivors with substantial morbidity (ie, ≥10 CHCs of grade 1-4 or ≥3 CHCs of grade 3-4) were at increased risk of death as early as 2 years after baseline assessment. Work from other researchers^31^ supports these findings, suggesting that self-reported cardiovascular risk factors and unhealthy lifestyle were associated with a 2-fold or higher increased risk of late health-related death. These data identified high-risk groups who may benefit from more frequent care, which will be important to consider for risk-stratified survivorship care approaches.^32^

Census block–level ADI was independently associated with an increased risk of late death, despite adjustment for modifiable CHCs, treatment exposures, demographic characteristics, and individual socioeconomic factors. Specifically, survivors living in the uppermost disadvantaged Census blocks were at a 5-fold to 8-fold increased risk of all-cause death compared with those living in the least disadvantaged Census block. In the general population, socioeconomic deprivation has been associated with all-cause and cardiovascular mortality.^32^ Similarly, the ADI has recently been incorporated into survival estimates in frontline pediatric treatment studies.^33,34^ To our knowledge, our cohort study is the first to evaluate the association between ADI and late mortality in childhood cancer survivors.

These findings have important public health implications. For example, use of the ADI has been proposed to identify patients, using physical address alone, who are returning to the most challenging socioeconomic environments and who may benefit from aggressive transitional care services.^35^ Such efforts should consider that survivors in disadvantaged SES neighborhoods may lack supportive resources (eg, access to transportation and medical services) to address health issues, potentially leading to increased risk of death. Moreover, living in disadvantaged neighborhoods may increase psychological stress, which has also been associated with higher risk of death.^36^ Efforts to mitigate risk factors associated with increased mortality in childhood cancer survivors may also be limited by food deserts and lack of green spaces, which are more commonly observed in disadvantaged regions. Therefore, identification of ADI as a factor associated with the risk of late death in survivors strengthens support for public health policies that will direct resources to such regions and facilitate a multipronged approach to risk mitigation.

The growing number of cancer survivors necessitates reconsideration of approaches to the delivery of structured high-quality care. One approach has been risk-stratified care, which uses treatment factors to identify survivors with the highest lifetime risk of specific chronic conditions and late death.^37^ This stratification remains relatively static and does not account for risk factors acquired after cancer diagnosis and treatment, such as those identified in our study. By focusing on high-yield conditions and socioeconomic factors readily suitable for both individual- and health policy–level intervention, we identified survivors with the highest short-interval risk of late death. These groups should be prioritized for more frequent follow-up and intervention studies seeking to understand whether treatments and interventions used for similar conditions in the general population will have similar benefits and potentially reduce mortality in cancer survivors. By manually adjudicating causes of death in the context of death certificates, clinical documentation, and personal communication, we found consistencies between the NDI and our manually determined causes of death, supporting the ongoing use of NDI data for mortality analyses, particularly in cancer survivors.

### Limitations

This study has several limitations. First, some causes of death occurred too infrequently for cause-specific risk factor analysis. We were also unable to assign a Census block to all individuals. While we observed an association between unassigned ADI and significant increases in late mortality, we could not determine what implications accurate assignment of these individuals would have had for other risk estimates. In addition, we assumed select CHCs were modifiable or could be responsive to intervention based on the availability of beneficial treatments and interventions for similar conditions in the general population; however, the benefits of treatments and interventions and whether they would mitigate late mortality in cancer survivors remain unproven. Many individuals died before they could participate in the SJLIFE study and were not included in risk factor analyses. An ideal study design would involve longitudinal assessments beginning at cancer diagnosis, allowing evaluation of the association of risk factors with subsequent mortality at different time points over survivorship. We hypothesize that individuals who died before SJLIFE participation would have experienced increased risk given that they likely experienced more aggressive disease and greater treatment-related toxic effects.

## Conclusions

In this longitudinal cohort study, modifiable CHCs and neighborhood-level socioeconomic factors were associated with increased all-cause and cause-specific late mortality in childhood cancer survivors after adjustment for personal demographic, treatment, and socioeconomic factors. Investigations seeking to mitigate these factors will be important to improving health outcomes and developing risk-stratification strategies to optimize care delivery to survivors at varying risk of adverse health events. Studies addressing these risk factors should design interventions deliverable within the constructs of available resources, and health policies aimed at deconstructing barriers inherent to disadvantaged regions are necessary to reduce risk for this population of patients with medically complex conditions.

## Notes

Supplement 1. **eTable 1.** Specific Criteria Used to Define Frailty in the St Jude Lifetime Cohort **eTable 2.** Baseline Modifiable Chronic Health Conditions and Lifestyle Factors Among Adult St. Jude Lifetime Cohort Participants With Baseline Assessments (n = 3407) by Vital Status **eTable 3.** Frequency of Deaths by Cause Among Survivors Eligible for the St Jude Lifetime Cohort (n = 9440) **eTable 4.** Mortality Rates (Deaths/1000 Person-Years) by Cause of Death Among All Eligible St Jude Lifetime Cohort Survivors by Every 5-Year Period Since Childhood Cancer Diagnosis **eTable 5.** All-Cause and Cause-Specific Standardized Mortality Ratios (SMRs) and 95% CIs Among Eligible St Jude Lifetime Cohort Survivors (n = 9440) by Sex and Childhood Cancer Diagnosis **eTable 6.** Multivariable Associations Between Modifiable Risk Factors, Including Specific Chronic Health Conditions and Postbaseline All-Cause and Cause-Specific Mortality Rates in Adult St Jude Lifetime Cohort Participants With an On-Campus Assessment **eTable 7.** Discrepant Cases Between National Death Index and St Jude Children’s Research Hospital Cause of Death Assignment

Supplement 2.
Data Sharing Statement
