# Supplementary material for: Association of Modifiable Health Conditions and Social Determinants of Health With Late Mortality in Survivors of Childhood Cancer
Source: JAMA Netw Open. 2023 Feb 10;6(2):e2255395. doi: 10.1001/jamanetworkopen.2022.55395 (PMC9918884; doi:10.1001/jamanetworkopen.2022.55395)
Supplement: Supplement 1. — eTable 1. Specific Criteria Used to Define Frailty in the St Jude Lifetime Cohort eTable 2. Baseline Modifiable Chronic Health Conditions and Lifestyle Factors Among Adult St. Jude Lifetime Cohort Participants With Baseline Assessments (n = 3407) by Vital Status eTable 3. Frequency of Deaths by Cause Among Survivors Eligible for the St Jude Lifetime Cohort (n = 9440) eTable 4. Mortality Rates (Deaths/1000 Person-Years) by Cause of Death Among All Eligible St Jude Lifetime Cohort Survivors by Every 5-Year Period Since Childhood Cancer Diagnosis eTable 5. All-Cause and Cause-Specific Standardized Mortality Ratios (SMRs) and 95% CIs Among Eligible St Jude Lifetime Cohort Survivors (n = 9440) by Sex and Childhood Cancer Diagnosis eTable 6. Multivariable Associations Between Modifiable Risk Factors, Including Specific Chronic Health Conditions and Postbaseline All-Cause and Cause-Specific Mortality Rates in Adult St Jude Lifetime Cohort Participants With an On-Campus Assessment eTable 7. Discrepant Cases Between National Death Index and St Jude Children’s Research Hospital Cause of Death Assignment [file jamanetwopen-e2255395-s001.pdf]

Supplemental Online Content

Ehrhardt MJ, Liu Q, Dixon SB, et al. Association of modifiable health conditions and social determinants of health with late mortality in survivors of childhood cancer. *JAMA Netw Open*. 2023;6(2):e2255395. doi:10.1001/jamanetworkopen.2022.55395

eTable 1. Specific Criteria Used to Define Frailty in the St Jude Lifetime Cohort

eTable 2. Baseline Modifiable Chronic Health Conditions and Lifestyle Factors Among Adult St. Jude Lifetime Cohort Participants With Baseline Assessments (n = 3407) by Vital Status

eTable 3. Frequency of Deaths by Cause Among Survivors Eligible for the St Jude Lifetime Cohort (n = 9440)

eTable 4. Mortality Rates (Deaths/1000 Person-Years) by Cause of Death Among All Eligible St Jude Lifetime Cohort Survivors by Every 5-year Period Since Childhood Cancer Diagnosis

eTable 5. All-Cause and Cause-Specific Standardized Mortality Ratios (SMRs) and 95% CIs Among Eligible St Jude Lifetime Cohort Survivors (n = 9440) by Sex and Childhood Cancer Diagnosis

eTable 6. Multivariable Associations Between Modifiable Risk Factors, Including Specific Chronic Health Conditions and Postbaseline All-Cause and Cause-Specific Mortality Rates in Adult St Jude Lifetime Cohort Participants With an On-Campus Assessment

eTable 7. Discrepant Cases Between National Death Index and St Jude Children's Research Hospital Cause of Death Assignment

This supplemental material has been provided by the authors to give readers additional information about their work.

© 2023 Ehrhardt MJ et al. *JAMA Network Open*.

eTable 1. Specific Criteria Used to Define Frailty in the St Jude Lifetime Cohort

Frailty Component [a]	St. Jude Lifetime Cohort Criteria
Low lean muscle mass	Lean muscle mass by dual x-ray absorptiometry ≤ -1.5 age and sex specific SDS when compared to data from a national sample (National Health and Nutrition Examination Survey [NHANES]) [b]
Self-reported exhaustion	Score ≤ 40 (1 SDS, based on a standard normal distribution, this represents approximately the lowest 6.7% of the general population) on the Vitality Subscale of the Medical Outcomes Survey Short Form 36 (SF-36) [c]
Low-energy expenditure	Expended < 383 Kcal per week (males) or < 270 Kcal per week (females) during Leisure Time Physical Activity based on the NHANES Physical Activity Questionnaire [d]
Slowness	Females < 159 and males < 173 centimeters tall were classified as slow if they took ≥ 7 seconds, and females ≥ 159 and males ≥ 173 centimeters tall were classified as slow if they took ≥ 6 seconds to walk 15 feet at their usual pace
Weakness	Hand grip strength stratified by body mass index and sex

Males		Females	
**BMI**	**Cut point**	**BMI**	**Cut point**
≤ 24 kg/m^2	≤ 29 kg	≤ 23 kg/m^2	≤ 17 kg
24.1 to 26 kg/m^2	≤ 30 kg	23.1 to 26 kg/m^2	≤ 17.3 kg
26.1 to 28 kg/m^2	≤ 30 kg	26.1 to 29 kg/m^2	≤ 18 kg
> 28 kg/m^2	≤ 32 kg	> 29 kg/m^2	≤ 21 kg

a. Fried LP, Tangen CM, Walston J, et al: Frailty in older adults: evidence for a phenotype. J Gerontol A Biol Sci Med Sci 56:M146-56, 2001
b. Kelly TL, Wilson KE, Heymsfield SB: Dual energy X-Ray absorptiometry body composition reference values from NHANES. PLoS One 4:e7038, 2009
c. Ware JE, Jr., Sherbourne CD: The MOS 36-item short-form health survey (SF-36). I. Conceptual framework and item selection. Med Care 30:473-83, 1992
d. National Health and Nutrition Examination Survey, 2005 - 2006 Data Documentation, Codebook, and Frequencies Physical Activity Monitor (PAXRAW_D). http://www.cdc.gov/nchs/nhanes/nhanes2005-2006/PAXRAW_D.htm

© 2023 Ehrhardt MJ et al. *JAMA Network Open*.

eTable 2. Baseline Modifiable Chronic Health Conditions and Lifestyle Factors Among Adult St. Jude Lifetime Cohort Participants With Baseline Assessments (n = 3407) by Vital Status

**Characteristics**	**Total N=3,407**		**Alive N=3,284**		**Dead N=123**		**P value**
	**N**	**(%)**	**N**	**(%)**	**N**	**(%)**	
**Frailty**							<.001
No	2,280	(69)	2,223	(70)	57	(48)	
Prefrail	721	(22)	686	(22)	35	(30)	
Frail	285	(9)	259	(8)	26	(22)	
**Healthy lifestyle index score**							0.002
0	179	(5)	165	(5)	14	(11)	
1	670	(20)	639	(20)	31	(25)	
2	1,275	(38)	1,229	(38)	46	(38)	
3	967	(29)	939	(29)	28	(23)	
4	266	(8)	263	(8)	3	(2)	
**Chronic condition grade**							
Dyslipidemia [a]							0.01
0	2,229	(65)	2,151	(65)	78	(63)	
1	688	(20)	672	(20)	16	(13)	
2	388	(11)	367	(11)	21	(17)	
3	93	(3)	86	(3)	7	(6)	
4	9	(0)	8	(0)	1	(1)	
Hypertension							<.001
0	1,535	(45)	1,509	(46)	26	(21)	
1	1,183	(35)	1,151	(35)	32	(26)	
2	523	(15)	483	(15)	40	(33)	
3	166	(5)	141	(4)	25	(20)	
Diabetes							<.001
0	2,671	(78)	2,606	(79)	65	(53)	
1	448	(13)	413 (13)		35	(28)	
2	170	(5)	156	(5)	14	(11)	
3	118	(3)	109	(3)	9	(7)	
Underweight/Obesity [a,b]							0.22
0	1,209	(35)	1,169	(36)	40	(33)	
2	1,049	(31)	1,006	(31)	43	(35)	
3	910	(27)	883	(27)	27	(22)	
4	239	(7)	226	(7)	13	(11)	
Bone mineral deficiency							0.02
0	1,949	(57)	1,875	(57)	74	(60)	
1	1,106	(32)	1,077	(33)	29	(24)	
2	352	(10)	332	(10)	20	(16)	
Hypogonadism							<.001
0	2,513	(74)	2,457	(75)	56	(46)	
1	324	(10)	301	(9)	23	(19)	
2	339	(10)	316	(10)	23	(19)	
3	231	(7)	210	(6)	21	(17)	
Hypothyroidism							0.001
0	2,688	(79)	2,607	(79)	81	(66)	
1	23	(1)	23	(1)	0	(0)	
2	694	(20)	652	(20)	42	(34)	
3	2	(0)	2	(0)	0	(0)	

© 2023 Ehrhardt MJ et al. *JAMA Network Open*.

Characteristics	Total N=3,407		Alive N=3,284		Dead N=123		P value
	N	(%)	N	(%)	N	(%)	
Adrenal insufficiency							0.91
0	3,210	(94)	3,093	(94)	117	(95)	
1	107	(3)	103	(3)	4	(3)	
2	87	(3)	85	(3)	2	(2)	
3	3	(0)	3	(0)	0	(0)	

[a] Only dyslipidemia and underweight/obesity include a grade 4
[b] Underweight/Obesity does not include a grade 1

© 2023 Ehrhardt MJ et al. *JAMA Network Open.*

eTable 3. Frequency of Deaths by Cause Among Survivors Eligible for the St Jude Lifetime Cohort (n = 9440)

Causes	ICD-10 Code	ICD-9 Code	Total Deaths		Male		Female		Participants [a]		Non-participants	
			N	%	N	%	N	%	N	%	N	%
**Total**			**1,281**		**770**		**511**		**155**		**1,126**	
**Recurrence**			**492**	**38.4**	**307**	**39.9**	**185**	**36.2**	**5**	**3.2**	**487**	**43.3**
**Subsequent neoplasms**			**353**		**184**		**169**		**63**		**290**	
Oropharyngeal and gastrointestinal malignancies	C00-C25	140-157	47	3.7	32	4.2	15	2.9	12	7.7	35	3.1
Laryngeal, tracheal and Lung malignancies	C32-34	161-162	8	0.6	5	0.6	3	0.6	0	0.0	8	0.7
Malignancies of the skin	C43-44	172-173	1	0.1	1	0.1	0	0.0	0	0.0	1	0.1
Breast cancer	C50	174-175	19	1.5	0	0.0	19	3.7	4	2.6	15	1.3
Genitourinary malignancies	C51-58, C60-68	179-189	13	1.0	4	0.5	9	1.8	4	2.6	9	0.8
CNS malignancies	C70-C72	191-192	79	6.2	42	5.5	37	7.2	8	5.2	71	6.3
Hodgkin's disease	C81	201	7	0.5	5	0.6	2	0.4	3	1.9	4	0.4
Non-Hodgkin's lymphoma	C82-C85	200,202	11	0.9	7	0.9	4	0.8	6	3.9	5	0.4
Leukemia	C91-C95	204-208	51	4.0	33	4.3	18	3.5	2	1.3	49	4.4
Malignant neoplasm of bone, articular cartilage, or other connective and soft tissue	C40, C41, C49	170, 171	43	3.4	19	2.5	24	4.7	5	3.2	38	3.4
Benign meningioma	D32	225.2 and 225.4	6	0.5	2	0.3	4	0.8	3	1.9	3	0.3
All other malignant and other neoplasms	C26-C31, C37-39, C45-48, C69, C73-C80, C88, C90, C96 excluding C96.9, C97, D010, D126, D180, D332, D361, D469	158-160,163-165,173, 190,193-199, 203, 209, 212.5, 212.7, 225.1	46	3.6	23	3.0	23	4.5	13	8.4	33	2.9
Malignant and other neoplasms, NOS	C79.9, C80.0. C80.1, C96.9 D09.9, D36.9, D37-D44, D47-48	199.0, 199.1, 202.9 229.9, 235-237, 238.0-238.3, 238.79, 238.8, 238.9, 239	22	1.7	11	1.4	11	2.2	3	1.9	19	1.7
**Cardiac**			**72**		**50**		**22**		**20**		**52**	
Rheumatic and other valvular heart disease	I00-I09, add I34-I39	390-398, 424	5	0.4	3	0.4	2	0.4	1	0.6	4	0.4
Hypertensive heart disease	I11, I13	402, 404	2	0.2	1	0.1	1	0.2	1	0.6	1	0.1
Ischemic heart disease, includes myocardial infarction	I20-25	410-414, 429.2, 429.7	31	2.4	22	2.9	9	1.8	8	5.2	23	2.0
All other forms of heart disease	I26-I51, exclude I34-I39, and the 3 rows below	415-429.1, 429.3-429.9, exclude the 3 rows below, exclude 424 (now in casco=54), exclude 427 but with 427.5, exclude 429.7 which is above	5	0.4	4	0.5	1	0.2	.		5	0.4
Cardiomyopathy and heart failure	I50, I42-I43	425, 428	25	2.0	16	2.1	9	1.8	8	5.2	17	1.5
cardiac dysrhythmias	I47-I49	427 (excluding 427.5)	3	0.2	3	0.4	0	0.0	1	0.6	2	0.2

© 2023 Ehrhardt MJ et al. *JAMA Network Open*.

Causes	ICD-10 Code	ICD-9 Code	Total Deaths		Male		Female		Participants [a]		Non-participants	
			N	%	N	%	N	%	N	%	N	%
Heart disease, NOS	I51.89, I51.9	429.89, 429.9	1	0.1	1	0.1	0	0.0	1	0.6	0	0.0
**Pulmonary**			**44**		**31**		**13**		**8**		**36**	
Influenza and pneumonia	J09-J18	480-487	22	1.7	16	2.1	6	1.2	1	0.6	21	1.9
Chronic obstructive respiratory disease	J40-47	490-494, 496	7	0.5	7	0.9	0	0.0	4	2.6	3	0.3
Other respiratory diseases	J00-J06, J20-22, J30-J39, J60-J68, J70-J98,	034.0, 460-466, 470-478, 495, 500-506, 508-519	7	0.5	2	0.3	5	1.0	1	0.6	6	0.5
Pneumonitis due to solids and liquids	J69	507	4	0.3	3	0.4	1	0.2	0	0.0	4	0.4
Other interstitial lung disease, includes pulmonary fibrosis	J84	515, 516	4	0.3	3	0.4	1	0.2	2	1.3	2	0.2
**Other health-related**			**169**		**90**		**79**		**41**		**128**	
Other infectious causes of death	A00-B99 excluding the 4 rows below	001-139 and 771.3 excluding the 4 rows below	9	0.7	6	0.8	3	0.6	0	0	9	0.8
Septicemia	A40-A41	038	17	1.3	9	1.2	8	1.6	6	3.9	11	1.0
Viral hepatitis	B15-B19	070	8	0.6	6	0.8	2	0.4	4	2.6	4	0.4
Human immunodeficiency virus disease	B20-B24	042-044	0	0	0	0	0	0	0	0	0	0
Infectious cause of death, unspecified	B94.9, B99.9	136.9, 139.8	0	0	0	0	0	0	0	0	0	0
Diabetes mellitus	E10-E14	250	7	0.5	3	0.4	4	0.8	2	1.3	5	0.4
Essential (primary) hypertension and hypertensive renal disease	I10, I12	401,403	1	0.1	1	0.1	0	0.0	1	0.6	0	0.0
Cerebrovascular diseases	I60-I69	430-434,436-438	18	1.4	8	1.0	10	2.0	8	5.2	10	0.9
Other atherosclerotic and vascular disease	I70, I71-I78, I80-I99 excluding I99.9	440, 441-448, 451-459 excluding 459.9	6	0.5	3	0.4	3	0.6	4	2.6	2	0.2
Chronic liver disease and cirrhosis	K70, K73-K74	571	9	0.7	3	0.4	6	1.2	1	0.6	21	1.9
Renal failure	N17-N19	584-586	10	0.8	6	0.8	4	0.8	4	2.6	3	0.3
Complications of pregnancy, childbirth and the puerperium	O00-099	630-676	1	0.1	0	0.0	1	0.2	1	0.6	6	0.5
Congenital malformations, deformations, and chromosomal abnormalities	Q00-Q99	740-759	5	0.4	3	0.4	2	0.4	0	0.0	4	0.4
Symptoms, signs and abnormal clinical and laboratory findings, not elsewhere classified	R00-R99	780-799	11	0.9	5	0.6	6	1.2	2	1.3	2	0.2
All other diseases (Residual)	All other known codes	All other known codes	67	5.2	37	4.8	30	5.9	9	5.8	58	5.2
**External**			**139**		**104**		**35**		**18**		**121**	
All transport accidents, includes motor vehicle collision	V01-V99, Y85	E800-E848, E929.0, E929.1	44	3.4	32	4.2	12	2.3	2	1.3	42	3.7
Falls	W00-W19	E880-E888	4	0.3	3	0.4	1	0.2	1	0.6	3	0.3

© 2023 Ehrhardt MJ et al. *JAMA Network Open.*

Causes	ICD-10 Code	ICD-9 Code	Total Deaths		Male		Female		Participants [a]		Non-participants	
			N	%	N	%	N	%	N	%	N	%
Other and unspecified nontransport accidents and sequelae	W20-W64, W65-W99, X00-X09, X10-X39, X50-X59, Y86	E890-E909, E910-E924.0, E924.8-E928, E929.2-E929.9	14	1.1	11	1.4	3	0.6	2	1.3	12	1.1
Accidental poisoning and exposure to noxious substances	X40-X49	E850-E869, E924.1	21	1.6	15	1.9	6	1.2	7	4.5	14	1.2
Suicide	X60-X84, Y87	E950-E959	30	2.3	26	3.4	4	0.8	2	1.3	28	2.5
Homicide	X92-Y09, Y87.1	E960-E969	11	0.9	6	0.8	5	1.0	2	1.3	9	0.8
Other external causes of death	Y10-Y36, Y87.2, Y89.0, Y89.1, Y89.9,	E970-E978, E990-E999	5	0.4	5	0.6	0	0.0	1	0.6	4	0.4
Complications of medical and surgical care	Y40-Y84, Y88	E870-E879, E930-E949	10	0.8	6	0.8	4	0.8	1	0.6	9	0.8
Unknown			12	0.9	4	0.5	8	1.6	0	0.0	12	1.1

[a] Includes pediatric and adult participants

© 2023 Ehrhardt MJ et al. *JAMA Network Open.*

eTable 4. Mortality Rates (Deaths/1000 Person-Years) by Cause of Death Among All Eligible St Jude Lifetime Cohort Survivors by Every 5-year Period Since Childhood Cancer Diagnosis

Time from diagnosis (Years)	All-cause		Recurrence		Health-related										External	
					All Health-related		Subsequent Neoplasm		Cardiac		Pulmonary		Other Health-related			
	Rate	95% CI	Rate	95% CI	Rate	95% CI	Rate	95% CI	Rate	95% CI	Rate	95% CI	Rate	95% CI	Rate	95% CI
5-9	13.0	11.9 - 14.1	9.2	8.3 - 10.1	3.1	2.6 - 3.6	1.9	1.5 - 2.4	0.1	0.0 - 0.2	0.2	0.1 - 0.4	0.8	0.6 - 1.1	0.7	0.4 - 1.0
10-14	5.7	4.9 - 6.6	2.1	1.7 - 2.7	2.8	2.3 - 3.5	2.0	1.6 - 2.6	0.1	0.0 - 0.3	0.2	0.0 - 0.4	0.6	0.3 - 0.9	0.7	0.4 - 1.1
15-19	5.4	4.5 - 6.4	0.8	0.5 - 1.3	3.4	2.7 - 4.2	1.9	1.4 - 2.5	0.4	0.2 - 0.7	0.3	0.1 - 0.6	0.8	0.5 - 1.2	1.1	0.7 - 1.6
20-24	5.7	4.7 - 6.9	0.5	0.3 - 1.0	3.8	3.0 - 4.8	2.3	1.7 - 3.1	0.6	0.3 - 1.0	0.2	0.0 - 0.5	0.8	0.4 - 1.3	1.3	0.8 - 1.9
25-29	6.1	4.8 - 7.5	0.4	0.2 - 0.9	4.6	3.6 - 5.9	2.2	1.5 - 3.1	0.7	0.3 - 1.3	0.3	0.1 - 0.7	1.4	0.9 - 2.2	0.9	0.5 - 1.6
30-34	9.1	7.2 - 11.3	0.3	0.1 - 1.0	7.2	5.6 - 9.2	2.8	1.9 - 4.2	1.1	0.5 - 2.0	0.9	0.4 - 1.7	2.4	1.5 - 3.6	1.4	0.8 - 2.4
35-39	16.5	13.1 - 20.5	0.2	0.0 - 1.1	14.7	11.5 - 18.5	6.8	4.7 - 9.6	3.0	1.7 - 5.0	0.8	0.2 - 2.1	4.0	2.5 - 6.2	1.4	0.6 - 2.9
40+	20.8	15.9 - 26.7	0.3	0.0 - 1.9	19.4	14.7 - 25.1	8.8	5.8 - 13.0	3.1	1.4 - 5.8	0.7	0.1 - 2.5	6.8	4.2 - 10.5	1.0	0.2 - 3.0
All years	8.6	8.1 - 9.1	3.3	3.0 - 3.6	4.3	3.9 - 4.6	2.4	2.1 - 2.6	0.5	0.4 - 0.6	0.3	0.2 - 0.4	1.1	1.0 - 1.3	0.9	0.8 - 1.1

© 2023 Ehrhardt MJ et al. *JAMA Network Open*.

eTable 5. All-Cause and Cause-Specific Standardized Mortality Ratios (SMRs) and 95% CIs Among Eligible St Jude Lifetime Cohort Survivors (n = 9440) by Sex and Childhood Cancer Diagnosis

	Deaths by Cause																				
				Health-related																	
**Characteristic**	**All-cause Mortality**			**All Health-related**			**Subsequent Neoplasms**			**Cardiac**			**Pulmonary**			**Other Health-related**			**External**		
	**N**	**SMR**	**95% CI**	**N**	**SMR**	**95% CI**	**N**	**SMR**	**95% CI**	**N**	**SMR**	**95% CI**	**N**	**SMR**	**95% CI**	**N**	**SMR**	**95% CI**	**N**	**SMR**	**95% CI**
**All patients**	1,281	7.6	7.2 - 8.1	638	7.6	7.0 - 8.2	353	16.0	14.4 - 17.8	72	4.2	3.3 - 5.3	44	9.0	6.5 - 12.0	169	4.3	3.7 - 5.0	139	1.6	1.4 - 1.9
**Sex**																					
Male	770	6.3	5.9 - 6.8	355	6.6	5.9 - 7.3	184	14.8	12.8 - 17.1	50	3.9	2.9 - 5.2	31	10.5	7.2 - 15.0	90	3.5	2.8 - 4.3	104	1.5	1.3 - 1.9
Female	511	11.0	10.1 - 12.0	283	9.6	8.5 - 10.7	169	17.5	14.9 - 20.3	22	4.9	3.1 - 7.5	13	6.6	3.5 - 11.3	79	5.8	4.6 - 7.3	35	2.1	1.4 - 2.9
**Diagnosis**																					
Leukemia																					
Acute lymphoblastic leukemia	303	6.2	5.5 - 6.9	145	6.1	5.1 - 7.2	97	15.5	12.6 - 18.9	10	2.1	1.0 - 3.9	9	6.4	2.9 - 12.2	29	2.5	1.7 - 3.7	26	1.0	0.7 - 1.5
Acute myeloid leukemia	38	7.9	5.6 - 10.8	20	9.4	5.7 - 14.5	8	14.9	6.4 - 29.4	2	5.0	0.6 - 18.0	3	23.5	4.7 - 68.7	7	6.5	2.6 - 13.5	6	2.2	0.8 - 4.9
Chronic myeloid leukemia	14	13.3	7.3 - 22.3	5	9.7	3.1 - 22.6	3	20.4	4.1 - 59.5	0	0.0	0.0 - 40.5	0	0.0	0.0 - 117.4	2	8.1	0.9 - 29.4	0	0.0	0.0 - 6.9
Other	2	13.8	1.5 - 49.8	2	33.1	3.7 - 119.6	0	0.0	0.0 - 260.3	0	0.0	0.0 - 299.1	0	0.0	0.0 - 1073.0	2	65.8	7.4 - 237.4	0	0.0	0.0 - 43.6
Central nervous system tumors																					
Astrocytoma	86	15.5	12.4 - 19.2	35	18.0	12.5 - 25.0	16	34.3	19.6 - 55.7	1	3.1	0.0 - 17.1	4	31.2	8.4 - 79.9	14	13.6	7.4 - 22.9	7	1.9	0.8 - 4.0
Medulloblastoma	54	17.8	13.3 - 23.2	18	16.5	9.8 - 26.1	16	62.4	35.7 - 101.4	0	0.0	0.0 - 18.5	0	0.0	0.0 - 53.6	2	3.5	0.4 - 12.8	2	1.0	0.1 - 3.7
Ependymoma	64	60.3	46.4 - 77.0	18	45.8	27.1 - 72.4	12	124.9	64.5 - 218.2	0	0.0	0.0 - 61.4	1	36.3	0.5 - 202.0	5	23.9	7.7 - 55.7	2	3.0	0.3 - 10.8
Craniopharyngioma	20	14.6	8.9 - 22.6	13	28.0	14.9 - 47.9	7	69.5	27.9 - 143.3	0	0.0	0.0 - 41.8	1	34.7	0.5 - 193.3	5	20.3	6.5 - 47.4	1	1.1	0.0 - 6.2
Other	13	18.4	9.8 - 31.5	7	31.7	12.7 - 65.4	2	38.9	4.4 - 140.6	0	0.0	0.0 - 112.6	0	0.0	0.0 - 236.8	5	41.3	13.3 - 96.5	0	0.0	0.0 - 7.6
Hodgkin lymphoma	185	7.2	6.2 - 8.4	137	9.3	7.8 - 11.1	68	16.8	13.1 - 21.4	32	10.1	6.9 - 14.3	10	12.1	5.8 - 22.2	27	4.1	2.7 - 5.9	20	1.8	1.1 - 2.8
Non-Hodgkin lymphoma	74	4.6	3.6 - 5.8	51	6.3	4.7 - 8.3	23	11.3	7.1 - 16.9	10	5.6	2.7 - 10.2	4	8.9	2.4 - 22.7	14	3.7	2.0 - 6.2	15	1.9	1.1 - 3.1
Renal	50	5.4	4.0 - 7.2	21	5.0	3.1 - 7.7	9	8.4	3.9 - 16.0	0	0.0	0.0 - 4.6	1	4.0	0.1 - 22.2	11	5.3	2.7 - 9.5	13	2.6	1.4 - 4.4
Neuroblastoma	73	12.0	9.4 - 15.1	16	6.1	3.5 - 9.9	11	16.5	8.2 - 29.5	0	0.0	0.0 - 7.6	1	6.2	0.1 - 34.5	4	3.1	0.8 - 7.8	8	2.3	1.0 - 4.6
Soft tissue sarcoma	77	6.9	5.5 - 8.6	37	6.7	4.7 - 9.3	20	14.2	8.6 - 21.9	6	5.2	1.9 - 11.2	0	0.0	0.0 - 11.7	11	4.2	2.1 - 7.5	9	1.6	0.7 - 3.0
Ewing sarcoma	66	10.1	7.8 - 12.8	23	6.2	3.9 - 9.3	5	5.0	1.6 - 11.7	5	6.0	1.9 - 14.0	1	4.6	0.1 - 25.6	12	7.3	3.7 - 12.7	9	3.2	1.4 - 6.0
Osteosarcoma	52	5.9	4.4 - 7.7	24	4.5	2.9 - 6.7	13	8.7	4.6 - 14.9	4	3.3	0.9 - 8.5	2	6.3	0.7 - 22.8	5	2.1	0.7 - 5.0	6	1.7	0.6 - 3.8
Retinoblastoma	30	6.6	4.4 - 9.4	24	11.3	7.3 - 16.9	21	38.2	23.6 - 58.4	0	0.0	0.0 - 9.5	0	0.0	0.0 - 28.4	3	2.9	0.6 - 8.4	3	1.2	0.2 - 3.6
Germ cell tumor	18	5.0	3.0 - 7.9	14	7.1	3.9 - 11.9	7	12.1	4.9 - 25.0	0	0.0	0.0 - 10.4	4	33.0	8.9 - 84.4	3	3.3	0.7 - 9.6	1	0.6	0.0 - 3.4
Liver malignancies	5	4.7	1.5 - 11.0	2	4.7	0.5 - 17.1	0	0.0	0.0 - 36.6	1	12.3	0.2 - 68.4	0	0.0	0.0 - 145.7	1	4.7	0.1 - 26.0	0	0.0	0.0 - 5.7
Melanoma	7	5.8	2.3 - 12.0	2	3.1	0.4 - 11.3	2	12.0	1.3 - 43.2	0	0.0	0.0 - 25.3	0	0.0	0.0 - 100.4	0	0.0	0.0 - 12.8	0	0.0	0.0 - 6.5
Nasopharyngeal carcinoma	15	9.4	5.2 - 15.4	8	8.7	3.8 - 17.2	3	12.1	2.4 - 35.4	0	0.0	0.0 - 18.5	3	58.7	11.8 - 171.4	2	4.8	0.5 - 17.3	5	7.3	2.4 - 17.0
Histiocytosis	7	2.0	0.8 - 4.1	3	1.7	0.3 - 5.1	1	2.2	0.0 - 12.3	0	0.0	0.0 - 10.1	0	0.0	0.0 - 36.5	2	2.5	0.3 - 8.9	3	1.6	0.3 - 4.8
Other	28	12.1	8.0 - 17.5	13	10.8	5.8 - 18.5	9	26.9	12.3 - 51.1	1	4.3	0.1 - 24.1	0	0.0	0.0 - 51.5	3	5.3	1.1 - 15.5	3	2.7	0.5 - 7.9

© 2023 Ehrhardt MJ et al. *JAMA Network Open*.

eTable 6. Multivariable Associations Between Modifiable Risk Factors, Including Specific Chronic Health Conditions and Postbaseline All-Cause and Cause-Specific Mortality Rates in Adult St Jude Lifetime Cohort Participants With an On-Campus Assessment

Variable	Death by Cause																	
				Health-related														
	All-cause			All Health-related			Health-related Excluding Subsequent Neoplasm			Subsequent Neoplasm			Other Health-related			External		
	RR	95% CI	P	RR	95% CI	P	RR	95% CI	P	RR	95% CI	P	RR	95% CI	P	RR	95% CI	P
**Age at diagnosis**																		
<5 (Ref)	-			-			-			-			-			-		
5-9	0.8	0.5 - 1.5	0.51	0.8	0.4 - 1.5	0.53	0.8	0.4 - 2.0	0.70	0.9	0.4 - 2.3	0.85	0.9	0.3 - 2.6	0.90	1.0	0.2 - 6.4	0.97
10-14	0.8	0.4 - 1.5	0.49	0.7	0.4 - 1.4	0.31	0.6	0.3 - 1.6	0.35	0.9	0.3 - 2.4	0.84	0.2	0.1 - 1.0	0.05	2.0	0.4 - 10.8	0.43
15-19	0.9	0.5 - 1.6	0.63	0.8	0.4 - 1.5	0.44	0.8	0.3 - 1.9	0.58	0.9	0.3 - 2.6	0.86	0.9	0.3 - 2.7	0.80	2.4	0.4 - 16.2	0.37
**Sex**																		
Male vs. Female (Ref)	**1.9**	**1.2 - 2.9**	**0.006**	**1.7**	**1.1 - 2.8**	**0.03**	**2.7**	**1.3 - 5.3**	**0.006**	1.1	0.5 - 2.3	0.77	1.9	0.8 - 4.5	0.17	**6.6**	**1.5 - 29.1**	**0.01**
**Race**																		
Non-White vs. White (Ref)	0.8	0.4 - 1.4	0.36	0.8	0.5 - 1.6	0.57	0.8	0.4 - 1.9	0.65	0.7	0.3 - 1.9	0.52	1.2	0.4 - 3.6	0.69	0.4	0.1 - 2.6	0.36
**Annual household income**																		
≥100K (Ref)	-			-			-			-			-			-		
60-99K	1.0	0.5 - 2.0	0.95	1.0	0.5 - 2.2	0.95	0.6	0.2 - 1.7	0.38	1.9	0.6 - 6.8	0.30	0.6	0.2 - 2.3	0.47	0.5	0.1 - 4.8	0.58
20-59K	1.0	0.5 - 1.9	0.91	1.0	0.5 - 2.1	0.99	0.6	0.2 - 1.5	0.26	2.2	0.7 - 7.4	0.19	0.4	0.1 - 1.5	0.19	0.7	0.1 - 5.1	0.75
<20K	0.7	0.3 - 1.6	0.42	0.8	0.3 - 2.1	0.68	0.5	0.2 - 1.9	0.32	1.6	0.4 - 6.5	0.54	0.3	0.1 - 1.8	0.20	0.2	0.0 - 2.6	0.20
Missing	1.2	0.5 - 2.9	0.62	1.3	0.5 - 3.3	0.60	1.6	0.5 - 5.4	0.42	0.7	0.1 - 4.4	0.71	1.4	0.3 - 6.2	0.63	0.5	0.0 - 5.6	0.55
**Health insurance status**																		
Private (Ref)	-			-						-			-			-		
None	1.3	0.7 - 2.3	0.39	1.1	0.6 - 2.1	0.81	0.9	0.4 - 2.3	0.85	1.4	0.5 - 3.5	0.53	0.6	0.2 - 2.1	0.44	4.0	0.8 - 19.4	0.09
Public	**2.8**	**1.6 - 4.7**	**<.001**	**2.4**	**1.4 - 4.3**	**0.002**	**2.5**	**1.1 - 5.7**	**0.03**	**2.5**	**1.1 - 5.8**	**0.03**	2.2	0.8 - 6.4	0.13	**6.8**	**1.2 - 38.3**	**0.03**
Missing	2.4	0.7 - 8.6	0.18	1.9	0.4 - 8.7	0.41	2.5	0.5 - 12.8	0.28	0.0	0.0 - 7.2	0.42	4.4	0.7 - 26.7	0.10	5.3	0.3 - 105.8	0.28
**Grade ≥2 modifiable chronic health conditions[a,b]**																		
**Hypertension**																		
Grade 1	1.2	0.7 - 2.0	0.58	1.1	0.6 - 2.0	0.82	1.5	0.6 - 3.8	0.41	0.7	0.3 - 1.7	0.47	1.0	0.3 - 3.3	0.99	2.3	0.5 - 9.4	0.26
Grade 2	**2.1**	**1.2 - 3.7**	**0.01**	**2.1**	**1.1 - 3.9**	**0.02**	**3.4**	**1.3 - 8.5**	**0.01**	1.4	0.6 - 3.3	0.50	2.3	0.7 - 7.2	0.17	0.8	0.1 - 5.8	0.82
Grade 3	**2.2**	**1.1 - 4.3**	**0.03**	1.9	0.9 - 3.9	0.11	**3.6**	**1.3 - 10.3**	**0.02**	0.7	0.2 - 2.4	0.52	3.1	0.8 - 12.1	0.10	3.0	0.3 - 32.2	0.36
**Diabetes**																		
Grade 1	1.1	0.7 - 1.8	0.68	1.1	0.7 - 1.9	0.62	0.9	0.4 - 1.8	0.73	1.7	0.7 - 3.8	0.21	0.8	0.3 - 2.2	0.71	0.8	0.2 - 4.4	0.81
Grade 2	1.2	0.6 - 2.4	0.69	1.5	0.7 - 3.2	0.31	1.4	0.5 - 3.8	0.50	1.5	0.4 - 5.3	0.53	1.7	0.5 - 5.9	0.44	0.0	0.0 - 3.2	0.20
Grade 3	**2.4**	**1.1 - 5.3**	**0.03**	**3.2**	**1.4 - 7.2**	**0.005**	2.4	0.7 - 8.5	0.18	**3.9**	**1.3 - 11.7**	**0.01**	**4.5**	**1.1 - 17.6**	**0.03**	0.0	0.0 - 2.9	0.16
**Dyslipidemia**																		
Grade 1	0.6	0.3 - 1.1	0.10	**0.4**	**0.2 - 0.9**	**0.03**	0.4	0.2 - 1.2	0.10	0.5	0.2 - 1.4	0.17	0.5	0.2 - 1.6	0.24	1.2	0.3 - 5.1	0.82
Grade 2	**0.6**	**0.3 - 1.0**	**0.04**	**0.5**	**0.3 - 0.8**	**0.01**	**0.3**	**0.1 - 0.7**	**0.005**	0.8	0.3 - 1.9	0.63	**0.2**	**0.1 - 0.7**	**0.008**	1.2	0.2 - 8.4	0.86
Grade 3-4	0.6	0.2 - 1.4	0.26	0.4	0.1 - 1.1	0.08	0.4	0.1 - 1.6	0.22	0.3	0.0 - 2.1	0.20	0.6	0.1 - 2.4	0.43	4.0	0.3 - 57.6	0.30
**Bone mineral deficiency**																		
Grade 2 vs. 0-1	1.3	0.7 - 2.4	0.34	1.0	0.5 - 2.1	0.89	1.1	0.4 - 3.1	0.78	0.8	0.3 - 2.2	0.68	1.2	0.4 - 3.9	0.76	3.6	0.7 - 17.8	0.12
**Hypogonadism**																		
Grade 1	1.5	0.8 - 2.6	0.19	1.8	1.0 - 3.3	0.07	1.2	0.5 - 2.9	0.64	**2.7**	**1.1 - 6.8**	**0.04**	1.8	0.6 - 5.9	0.32	0.0	0.0 - 3.0	0.35
Grade 2-3	1.6	1.0 - 2.6	0.05	1.6	1.0 - 2.8	0.06	1.7	0.8 - 3.5	0.17	1.6	0.7 - 3.5	0.24	3.4	1.3 - 8.7	0.01	2.3	0.5 - 9.3	0.26
**Hypothyroidism**																		
Grade 2-3 vs. 0-1	1.2	0.7 - 1.9	0.49	1.3	0.8 - 2.1	0.37	0.9	0.4 - 1.8	0.69	2.0	0.9 - 4.2	0.08	0.6	0.2 - 1.7	0.36	1.3	0.3 - 6.1	0.75
**Adrenal insufficiency**																		
Grade 1	1.4	0.5 - 4.5	0.54	2.1	0.6 - 7.1	0.21	4.0	0.7 - 23.7	0.12	1.4	0.3 - 7.4	0.68	2.2	0.2 - 23.3	0.50	0.0	0.0 - 2.1	0.13

© 2023 Ehrhardt MJ et al. *JAMA Network Open*.

Variable	Death by Cause																	
				Health-related														
	All-cause			All Health-related			Health-related Excluding Subsequent Neoplasm			Subsequent Neoplasm			Other Health-related			External		
	RR	95% CI	P	RR	95% CI	P	RR	95% CI	P	RR	95% CI	P	RR	95% CI	P	RR	95% CI	P
Grade 2-3	0.7	0.2 - 3.2	0.67	1.0	0.2 - 4.7	0.95	2.1	0.2 - 18.7	0.49	0.5	0.1 - 4.0	0.47	3.0	0.3 - 28.1	0.33	0.0	0.0 - 2.4	0.14
**Healthy lifestyle index[c]**																		
4 (Healthy in all 4; Ref)	-			-			-			-			-			-		
3	2.0	0.5 - 8.6	0.36	3.6	0.5 - 27.5	0.22	1.3	0.1 - 11.0	0.83	2.3	0.3 - 17.9	0.43	1.2	0.1 - 13.2	0.86	0.4	0.0 - 4.4	0.43
2	1.9	0.4 - 8.2	0.39	3.3	0.4 - 25.4	0.25	2.0	0.2 - 16.7	0.53	1.3	0.2 - 10.1	0.82	1.9	0.2 - 20.0	0.58	0.3	0.0 - 4.0	0.39
1	1.9	0.4 - 8.6	0.38	3.2	0.4 - 25.5	0.26	1.2	0.1 - 10.8	0.87	1.9	0.2 - 15.7	0.53	1.4	0.1 - 16.9	0.78	0.8	0.1 - 8.1	0.83
0 (Unhealthy in all 4)	2.9	0.6 - 13.6	0.18	4.9	0.6 - 40.4	0.14	2.2	0.2 - 20.3	0.50	2.3	0.2 - 22.1	0.46	4.9	0.4 - 56.5	0.20	0.2	0.0 - 6.3	0.37
**Area deprivation index**																		
1-10% (Ref)	-			-						-						-		
11-50%	3.4	0.8 - 14.7	0.10	6.8	0.9 - 51.7	0.06	- [c]			2.3	0.3 - 18.5	0.42	- [c]			0.3	0.0 - 4.1	0.39
51-80%	**5.1**	**1.2 - 21.9**	**0.03**	**8.7**	**1.1 - 66.5**	**0.04**	1.3	0.6 - 3.0	0.52	3.0	0.4 - 23.6	0.30	0.8	0.3 - 2.4	0.74	1.5	0.1 - 15.8	0.72
81-100%	**7.5**	**1.7 - 32.9**	**0.007**	**13.9**	**1.8 - 106.7**	**0.01**	**2.8**	**1.2 - 6.6**	**0.02**	3.8	0.5 - 31.7	0.21	2.7	1.0 - 7.5	0.05	2.0	0.2 - 23.8	0.57
Unassigned	**13.4**	**2.9 - 60.6**	**<.001**	**23.2**	**2.9 - 184.8**	**0.003**	**4.2**	**1.6 - 11.4**	**0.004**	7.4	0.8 - 66.2	0.07	1.8	0.4 - 7.6	0.44	3.2	0.2 - 62.9	0.45
**Frailty**																		
Not frail (Ref)	-			-			-			-			-			-		
Prefrail	1.5	0.9 - 2.4	0.10	**1.7**	**1.0 - 2.9**	**0.04**	1.7	0.8 - 3.5	0.17	2.0	1.0 - 4.2	0.06	1.6	0.6 - 4.0	0.33	0.6	0.1 - 3.4	0.53
Frail	**2.2**	**1.3 - 3.8**	**0.006**	**2.4**	**1.3 - 4.3**	**0.006**	**3.4**	**1.5 - 7.7**	**0.004**	1.6	0.6 - 4.4	0.33	2.4	0.8 - 7.2	0.12	4.2	0.9 - 19.8	0.07
**Treatment**																		
**Alkylators (mg/m^2)**																		
None	-			-			-			-			-			-		
<8000	1.0	0.6 - 1.8	0.96	0.9	0.5 - 1.7	0.70	0.5	0.2 - 1.3	0.15	1.5	0.6 - 4.0	0.39	0.3	0.1 - 1.0	0.06	2.5	0.5 - 11.6	0.24
≥8000	0.9	0.5 - 1.5	0.57	0.9	0.5 - 1.5	0.63	0.7	0.3 - 1.5	0.34	1.2	0.5 - 2.7	0.73	0.5	0.2 - 1.5	0.23	0.5	0.1 - 3.4	0.47
**Anthracyclines (mg/m^2)**																		
None	-			-			-			-			-			-		
0.1-<250	0.8	0.5 - 1.4	0.49	0.7	0.4 - 1.2	0.19	0.7	0.3 - 1.5	0.34	0.6	0.3 - 1.3	0.21	0.9	0.3 - 2.4	0.81	1.2	0.2 - 6.7	0.82
≥250	0.7	0.4 - 1.5	0.36	0.9	0.4 - 1.8	0.71	1.4	0.6 - 3.3	0.50	0.3	0.1 - 1.4	0.13	1.0	0.2 - 3.9	0.97	0.3	0.0 - 3.1	0.29
**Cranial irradiation (Gy)**																		
None	-			-			-			-			-			-		
<20	0.7	0.3 - 1.8	0.42	1.0	0.4 - 2.8	0.97	0.8	0.1 - 3.8	0.73	1.4	0.4 - 5.4	0.59	2.2	0.4 - 12.6	0.39	**0.0**	**0.0 - 0.5**	**0.01**
20-<30	0.7	0.4 - 1.3	0.23	0.9	0.5 - 1.7	0.80	0.7	0.3 - 1.8	0.49	1.2	0.5 - 3.1	0.66	1.7	0.6 - 4.9	0.29	**0.0**	**0.0 -0.6**	**0.02**
≥30	0.9	0.4 - 1.8	0.78	1.0	0.5 - 2.2	0.94	0.4	0.1 - 1.3	0.11	2.5	0.9 - 6.8	0.07	1.1	0.3 - 4.7	0.87	0.8	0.1 - 6.8	0.80
**Chest radiation (Gy)**																		
None	-			-			-			-			-			-		
<20	**2.3**	**1.0 - 5.4**	**0.046**	**2.6**	**1.1 - 6.5**	**0.04**	**6.0**	**1.9 - 19.0**	**0.002**	0.8	0.2 - 4.4	0.81	**5.2**	**1.3 - 21.4**	**0.03**	3.4	0.3 - 41.0	0.34
≥20	**3.2**	**2.0 - 5.0**	**<.001**	**3.7**	**2.3 - 6.2**	**<.001**	**7.2**	**3.6 - 14.7**	**<.001**	1.8	0.8 - 3.8	0.15	**7.2**	**2.8 - 18.3**	**<.001**	1.3	0.3 - 6.1	0.78

Adjusted for attained age as cubic splines; **Bolding indicates p<0.05**

[a] Excluding underweight/obesity due to inclusion in the healthy lifestyle index

[b] Smoking, drinking, exercise, and body mass index

[c] There were no deaths in the Area Deprivation Index 1-10% category, therefore 11-50% was used as the reference

© 2023 Ehrhardt MJ et al. *JAMA Network Open.*

eTable 7. Discrepant Cases Between National Death Index and St Jude Children's Research Hospital Cause of Death Assignment

**National Death Index Classification**	**St. Jude Children's Hospital Reclassification**
Other malignant neoplasms of other and unspecified sites (C76-C80, C97)	Malignant neoplasm of bone and articular cartilage of limbs
	Malignant neoplasm of bone and articular cartilage of other and unspecified sites
	Malignant neoplasm of brain
	Malignant neoplasm of breast
	Malignant neoplasm of liver and intrahepatic bile ducts (x2)
	Myeloid leukemia
	**Other medical procedures as the cause of abnormal reaction of the patient, or of later complication, without mention of misadventure at the time of the procedure**
	Secondary malignant neoplasm of respiratory and digestive organs
Other and unspecified leukemia (207-208)	Lymphoid leukemia
	Myeloid leukemia (x4)
	Neoplasm of uncertain behavior of other and unspecified sites and
	Secondary malignant neoplasm of other specified sites
	**Hemorrhage from respiratory passages**
	Malignant neoplasm of brain
	Myelodysplastic syndromes (x2)
Malignant neoplasms of brain (C71)	**Convulsions, not elsewhere classified**
	**Other complications of surgical and medical care, not elsewhere classified**
	**Other ill-defined and unspecified causes of mortality (x3)**
	**Pneumonia, organism unspecified**
	Secondary malignant neoplasm of other sites
Neoplasms of uncertain or unknown behavior of specified sites (D37-D47)	Malignant neoplasm of brain (x2)
	**Other complications of surgical and medical care, not elsewhere classified**
	**Other septicemia**
	**Viral pneumonia, not elsewhere classified**
	Malignant neoplasm of meninges
	Malignant neoplasm of peripheral nerves and autonomic nervous system
Myeloid leukemia (205, C92)	**Bacterial infection in conditions classified elsewhere and of**
	**Chronic liver disease and cirrhosis**
	Other malignant neoplasms of lymphoid and histiocytic tissue
	**Other interstitial pulmonary diseases**
Acute myocardial infarction (I21-I22)	Secondary malignant neoplasm of respiratory and digestive organs
All other and unspecified disorders of circulatory system (I83-I99)	Shock, not elsewhere classified
All other and unspecified viral diseases (A81, A85.0-A85.1, A85.8, A86-A89, B01, B04, B07-B09, B25-B34)	Influenza due to identified influenza virus
All other congenital anomalies (743-744,748-759)	**Bacterial infection in conditions classified elsewhere and of**
All other diseases of blood and blood-forming organs (286,288-289)	**Malignant neoplasm of bone and articular cartilage**
All other diseases of nervous system (G10-G12, G23-G25, G31, G36-G37, G43-G44, G47-G72, G81-G98)	**Malignant neoplasm of brain**
All other diseases of nervous system (G10-G12, G23-G25, G31, G36-G37, G43-G44, G47-G72, G81-G98)	**Other septicemia**
All other diseases of respiratory system (512, 514-519, J80-J84, J93-J98)	Pneumonia, organism unspecified
	**Malignant neoplasm of brain**
All other diseases of urinary system (591,593,595-599)	**Chronic liver disease and cirrhosis**
All other forms of chronic ischemic heart disease (I20, I25.1-I25.9)	**Hemorrhage from respiratory passages**
All other forms of chronic ischemic heart disease (I20, I25.1-I25.9)	**Intentional self-poisoning (suicide) by and exposure to other and unspecified drugs, medicaments, and biological substances**

© 2023 Ehrhardt MJ et al. *JAMA Network Open*.

All other infectious and parasitic diseases and late effects of other infectious and parasitic diseases (100-104, 130-136, 139)	Human immunodeficiency virus infection with specified conditions
Aplastic anemias (D60-D61)	**Other complications of surgical and medical care, not elsewhere classified**
	**Peritonitis**
Assault (homicide) by discharge of firearms (U01.4, X93-X95)	Discharge from other and unspecified firearms
Disorders of both mitral and aortic valves (I08.0)	**Postprocedural respiratory disorders, not elsewhere classified**
Hodgkin's disease (C81, 201)	**Cardiomyopathy**
	**Other septicemia**
	**Respiratory conditions due to other external agents**
	Malignant neoplasm of connective and other soft tissue
Influenza (J10-J11)	Other interstitial pulmonary diseases
Lymphoid leukemia (204, C91)	**Bronchopneumonia, organism unspecified**
	Myeloid leukemia
	**Cardiac arrest**
	Malignant immunoproliferative diseases
	Myeloid leukemia
	**Sequelae with surgical and medical care as external cause**
Malignant neoplasm of breast (C50)	**Heart failure**
Malignant neoplasm of eye and adnexa (C69)	Malignant melanoma of skin
Malignant neoplasms of all other and unspecified sites (195-199)	Malignant neoplasm of bone and articular cartilage
	Malignant neoplasm of retroperitoneum and peritoneum
Malignant neoplasms of bone and articular cartilage (170, C40-C41)	Secondary malignant neoplasm of other specified sites
	**Heart failure**
Malignant neoplasms of eye (190)	Malignant neoplasm of other endocrine glands and related structures
Malignant neoplasms of other and unspecified parts of nervous system (192)	Malignant neoplasm of brain
Malignant neoplasms of other mesothelial and soft tissue (C47-C49)	**Hemorrhage, not elsewhere classified**
	Malignant neoplasm of brain
	Malignant neoplasm of kidney, except renal pelvis
Malignant neoplasms of pharynx (C10-C13, C14.0)	**Other ill-defined and unspecified causes of mortality**
	**Respiratory conditions due to other external agents**
Malignant neoplasms of testis (C62)	Malignant neoplasm of brain
Malignant neoplasms of thyroid and other endocrine glands (C73-C75)	Malignant neoplasm of other connective and soft tissue
Malignant neoplasms of thyroid gland and other endocrine glands and related structures (193-194)	Malignant neoplasm of brain
Mycoses (110-118)	Bacterial infection in conditions classified elsewhere and of
Neoplasms of unspecified nature (239)	Malignant neoplasm of brain
	Malignant neoplasm of brain
Non-Hodgkin's lymphoma (C82-C85)	Malignant neoplasm of retroperitoneum and peritoneum
	**Other ill-defined and unspecified causes of mortality**
Other chronic obstructive pulmonary disease (J44)	**Sequelae with surgical and medical care as external cause**
Other complications of medical and surgical care and their sequelae (Y70-Y84, Y88.2-Y88.3)	Other septicemia
Other congenital malformations and deformations (Q10-Q18, Q35-Q89)	**Surgical operation and other surgical procedures as the cause of abnormal reaction of the patient, or of later complication, without mention of misadventure at the time of the procedure**
Other direct obstetric causes (642.0-642.3, 644-646, 651-659, 661-665, 667-669)	**Septicemia**
Other diseases of upper respiratory tract (J30-J39)	Bacterial pneumonia, not elsewhere classified
Other malignant neoplasms of lymphoid and histiocytic tissue (202)	Malignant neoplasm of connective and other soft tissue
Pneumonia due to other and unspecified organism (483,486)	**Malignant neoplasm of connective and other soft tissue**
Renal tubulo-interstitial diseases (N10-N15)	**Acute pancreatitis**
Septicemia (A40-A41)	**Secondary malignant neoplasm of other sites**
Stroke, not specified as hemorrhage or infarction (I64)	**Epilepsy**
Subarachnoid hemorrhage (I60)	**Malignant neoplasm of brain**
Viral hepatitis (B15-B19)	Chronic hepatitis, not elsewhere classified

© 2023 Ehrhardt MJ et al. *JAMA Network Open.*

Bolded cases reflect a major reclassification, rather than greater specification of causes of death

© 2023 Ehrhardt MJ et al. *JAMA Network Open.*
