# Supplementary material for: Association of Modifiable Health Conditions and Social Determinants of Health With Late Mortality in Survivors of Childhood Cancer
Source: JAMA Netw Open. 2023 Feb 10;6(2):e2255395. doi: 10.1001/jamanetworkopen.2022.55395 (PMC9918884; doi:10.1001/jamanetworkopen.2022.55395)
Supplement: Supplement 2. — Data Sharing Statement [file jamanetwopen-e2255395-s002.pdf]

Data Sharing Statement

Ehrhardt. Association of Modifiable Health Conditions and Social Determinants of Health With Late Mortality in Survivors of Childhood Cancer. *JAMA Netw Open*. Published February 10, 2023. doi:10.1001/jamanetworkopen.2022.55395

Data
Data available: Yes
Data types: Deidentified participant data
How to access data: Deidentified data will be available in aggregate on the St. Jude Cloud at time of publication (https://www.stjude.cloud/) and in detail upon request.
When available: With publication

Supporting Documents
Document types: None

Additional Information
Who can access the data: The Cloud data are available to all. Detailed data will be available to researchers whose proposed use of the data has been approved
Types of analyses: For any research purpose.
Mechanisms of data availability: With a signed data access agreement.
